# Cell-Cycle Modulation of Transcription Termination Factor Sen1

**DOI:** 10.1016/j.molcel.2018.03.010

**Published:** 2018-04-19

**Authors:** Hannah E. Mischo, Yujin Chun, Kevin M. Harlen, Brendan M. Smalec, Somdutta Dhir, L. Stirling Churchman, Stephen Buratowski

**Affiliations:** 1 Department of Biological Chemistry and Molecular Pharmacology, Harvard Medical School, Boston, MA 02115, USA; 2 Department of Genetics, Harvard Medical School, Boston, MA 02115, USA; 3 Sir William Dunn School of Pathology, Oxford University, South Parks Road, Oxford OX1 3RE, UK; 4 Mechanisms of Transcription Laboratory, Clare Hall Laboratories, Cancer Research UK London Research Institute, South Mimms EN6 3LD, UK

**Keywords:** Sen1, transcription termination, non-coding RNA, Nrd1-Nab3-Sen1, NNS, cell cycle, ubiquitin-proteasome system

## Abstract

Many non-coding transcripts (ncRNA) generated by RNA polymerase II in *S. cerevisiae* are terminated by the Nrd1-Nab3-Sen1 complex. However, Sen1 helicase levels are surprisingly low compared with Nrd1 and Nab3, raising questions regarding how ncRNA can be terminated in an efficient and timely manner. We show that Sen1 levels increase during the S and G2 phases of the cell cycle, leading to increased termination activity of NNS. Overexpression of Sen1 or failure to modulate its abundance by ubiquitin-proteasome-mediated degradation greatly decreases cell fitness. Sen1 toxicity is suppressed by mutations in other termination factors, and NET-seq analysis shows that its overexpression leads to a decrease in ncRNA production and altered mRNA termination. We conclude that Sen1 levels are carefully regulated to prevent aberrant termination. We suggest that ncRNA levels and coding gene transcription termination are modulated by Sen1 to fulfill critical cell cycle-specific functions.

## Highlights

• Transcription termination factor Sen1 levels fluctuate throughout the cell cycle
• APC targets Sen1 for degradation during G1
• Reduced Sen1 levels lower efficiency of Sen1-mediated termination
• Sen1 overexpression reduces cell viability because of excessive termination

## Graphical Abstract

**Figure:**
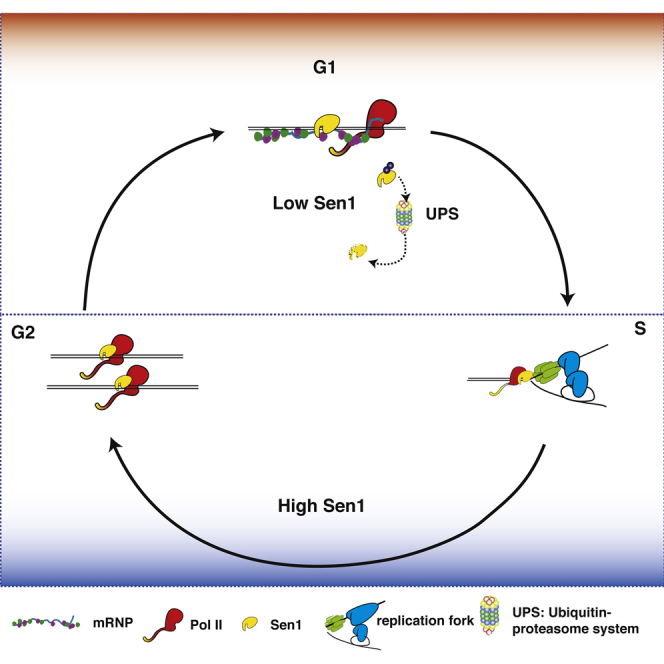

## Teaser

Transcription termination of noncoding RNAs by the Nrd1-Nab3-Sen1 (NNS) complex is affected by Sen1 protein levels. Controlled Sen1 degradation during the G1 phase of the cell cycle reduces NNS termination efficiency. In contrast, increased Sen1 levels lead to overactive noncoding RNA termination and reduced cell viability.

## Introduction

Genome-wide studies have unearthed a vast array of non-coding RNAs (ncRNAs) and aberrant transcripts that are mostly unstable and degraded in proximity to their transcription site (Jacquier, 2009, Wyers et al., 2005). Many of these transcripts arise through opportunistic transcription initiation events from nucleosome-depleted regions (NDRs) or result from failed 3′ end processing of coding transcripts (Pelechano et al., 2013, Rondón et al., 2009).

In *S. cerevisiae*, a large fraction of ncRNA is terminated by a specialized mechanism employing Nrd1-Nab3-Sen1 (NNS), which is distinct from the polyadenylation-coupled termination mechanism used for mRNA (Steinmetz et al., 2001). At ncRNAs, Nrd1-Nab3 heterodimers associate with the RNA polymerase II (Pol II) C-terminal domain (CTD), positioned to recognize short RNA sequence elements (GUA(A/G) for Nrd1 and UCUU(G) for Nab3) (Carroll et al., 2004, Carroll et al., 2007, Porrua et al., 2012) and to recruit the superfamily I helicase Sen1. Sen1 consequently disengages Pol II from the DNA template (Martin-Tumasz and Brow, 2015, Porrua and Libri, 2013). By remaining bound to RNA, Nrd1 can recruit the exosome to degrade many NNS terminated transcripts (Vanácová et al., 2005, Vasiljeva and Buratowski, 2006, Wyers et al., 2005). Overall, RNA degradation mutants have allowed detection of at least 6,000 ncRNAs in baker’s yeast (Mischo and Proudfoot, 2013), but functions have only been assigned to a fraction of these.

The 3′ end processing and termination of mRNA in *S. cerevisiae* requires the multi-protein cleavage and polyadenylation factor (CPF), comprised of three sub-complexes. Cleavage factors IA and IB (CFIA/B) recognize the RNA sequences specifying polyadenylation, leading to recruitment of CPF, which cleaves the pre-mRNA at the poly(A) site (PAS) and initiates polyadenylation. Cleavage generates a new uncapped 5′ RNA end onto which the exonuclease Rat1 loads to degrade the downstream transcript and release elongating Pol II (Fong et al., 2015, Kim et al., 2004, West et al., 2004).

Both termination pathways are connected through APT (associated with Pta1), a sub-complex associated with about half of the cellular CPF pool. APT is thought to modulate CPF activity and is required for the termination of many NNS substrates (reviewed in Mischo and Proudfoot, 2013). In addition to ncRNA termination, NNS also regulates the expression of some 42–305 mRNA genes by attenuation (Arigo et al., 2006, Creamer et al., 2011, Jamonnak et al., 2011, Schulz et al., 2013). Finally, on highly transcribed mRNA genes, NNS acts as a “failsafe” termination pathway for Pol II molecules that read through a PAS (Rondón et al., 2009, Webb et al., 2014). Overall, NNS restricts inadvertent transcription and controls gene expression through termination.

The cellular abundance of Nrd1 and Nab3 is estimated somewhat above that of RNA Pol II (Nrd1, 550–20,000; Nab3, 2,000–6,000; Pol II, 600–1,000) (Chong et al., 2015, Ghaemmaghami et al., 2003, Kulak et al., 2014, Newman et al., 2006). In contrast, the levels of Sen1, the enzymatic component of NNS, are well below Nrd1-Nab3 (64–500). This low copy number may suggest that Sen1 shuttles between various Nrd1-Nab3 heterodimers already bound to nascent RNA, effectively awaiting Sen1 to complete transcription termination. In addition, Sen1 may have functions outside of NNS because *SEN1* mutation results in aberrant nucleolar organization, genome instability, and replication defects (Alzu et al., 2012, Mischo et al., 2011, Ursic et al., 1995, Ursic et al., 2004).

Given such widespread cellular demand for Sen1 action, it appears surprising that its levels are kept low by proteasomal degradation (DeMarini et al., 1995). We therefore speculated that Sen1 levels might be adjusted to cellular demand, which might increase at certain points during the cell cycle; for example, when transcription encounters replication in S phase. To test this hypothesis, we monitored Sen1 abundance throughout the cell cycle and found that it increases in the S and G2 phases. We show that the ubiquitin-proteasome system degrades Sen1 preferentially during G1.

Cell cycle-specific E3 ubiquitin ligases of the ubiquitin-proteasome system ensure directional flow through the cell cycle (Finley et al., 2012, Sivakumar and Gorbsky, 2015) by marking ubiquitin-proteasome system substrates for timely degradation. During metaphase, the multi-subunit ubiquitin ligase anaphase-promoting complex (APC) binds its adaptor Cdc20 to degrade Pds1/Securin. This triggers anaphase and APC association with its alternative adaptor Cdh1. APC^Cdh1^ regulates entry into S phase by keeping S phase cyclins low. Although APC can have substrates with functions outside of cell cycle control (Menzel et al., 2014, Ostapenko et al., 2012), G1-specific degradation of a general transcription termination factor required in all phases of the cell cycle is unexpected. We find that, when Sen1 degradation is perturbed, ncRNA abundance and mRNA termination efficiency are substantially affected, and cell death is provoked. This argues that control of Sen1 levels and RNA termination throughout the cell cycle are critical.

## Results

### Sen1 Protein Levels and Activity Fluctuate throughout the Cell Cycle

**Figure 1:**
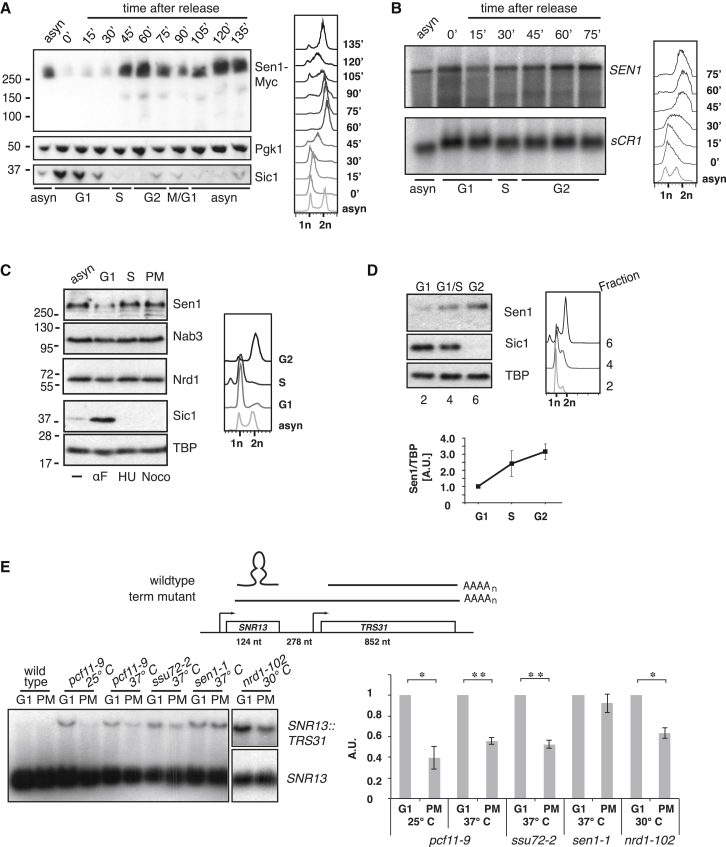
Sen1 Protein Levels Fluctuate throughout the Cell Cycle (A) Cells were αF-arrested and released into the cell cycle for the indicated time (see FACS analysis, right). Levels of C-terminally tagged Sen1-Myc (9E11), Sic1, and Pgk1 were analyzed by immunoblotting (left). (B) RNA analysis of *SEN1* and *sCR1.* RNA was prepared from cells grown as in (A) (see FACS analysis, right), and 10 μg was separated on a 1% agarose gel for RNA blotting (left). (C) Sen1 expression in drug-arrested cells. Cells grown in yeast extract, peptone, and dextrose (YPD) were arrested in G1 (5 μg/mL αF), S phase (200 mM hydroxyurea [HU]), or prometaphase (PM, 15 μg/mL nocodazole, see FACS analysis, right). Extract equivalent to 0.5 × 10^7^ cells (Nrd1) or 2 × 10^7^ cells (TBP, Sic1, Nab3, and Sen1 [antibody against the N terminus]) was analyzed by immunoblotting (left). (D) Sen1 expression in elutriated cells. Cells grown in YPD were separated by elutriating centrifugation and analyzed by FACS. Extracts prepared from fractions with G1, S, and G2 DNA content were analyzed by immunoblotting for Sen1-Myc (9E11), TBP, and Sic1. Quantification of three independent elutriations normalized to G1 levels with SEM is shown below. (E) RNA blot analysis of *SNR13* and *SNR13::TRS31* RNA. Wild-type, *pcf11-9*, *ssu72-2*, and *sen1-1* cells grown in YPD at a permissive temperature (25°C), were arrested with αF or nocodazole and shifted to a non-permissive temperature (37°C) for 30 min. *nrd1-102* cells were grown in YPD at 30°C before arrest with αF or nocodazole. 15 μg RNA was separated on a 1% agarose gel and analyzed by RNA blotting (left) against *SNR13* to detect the mature snoRNA and the *SNR13::TRS31* termination readthrough transcript (schematized above). The readthrough-to-snoRNA ratio for three to four biological replicates was normalized to the G1 value (y axis, SEM). Statistical significance of the difference between G1 and PM was calculated using Students’ t test. ^∗^p < 0.05, ^∗∗^p < 0.01.

To monitor Sen1 abundance over the cell cycle, cells expressing C-terminally Myc-tagged Sen1 were synchronized by alpha-factor (αF) arrest in late G1. After release, samples were taken every 15 min over a 2-hr time course and processed for immunoblotting and fluorescence-activated cell sorting (FACS) analysis (Figure 1A). In whole-cell extracts, Sen1 levels are reduced in G1 and increase toward S/G2, a pattern opposite to the G1-expressed Cdc28 inhibitor Sic1. This 10-fold difference in protein levels relative to αF arrest (Figure S1A) is primarily post-transcriptional because *SEN1* mRNA increases less than 2-fold in G2 (Figure 1B).

We excluded the possibility that Sen1 reduction in G1 reflects C-terminal partial proteolysis by monitoring Sen1 levels with an antibody raised against its N terminus (Figure 1C). In drug-arrested cells, Sen1 abundance decreases in G1 (αF) and increases in prometaphase (PM) after nocodazole arrest. The G1 depletion is specific to Sen1 because neither Nrd1 nor Nab3 levels fluctuate markedly throughout the cell cycle. Again, the levels of *SEN1* mRNA isolated from arrested cells remain similar (Figure S1B).

Finally, to discount that αF treatment artifactually causes Sen1 reduction, *SEN1-Myc*-tagged cells were elutriated to separate cells with G1, S, or G2 phase DNA content (Figure 1D). Although constant levels of *SEN1* mRNA are seen in all stages (Figure S1C), Sen1 protein levels are reduced 3-fold in G1 cells.

If Sen1 is limiting within NNS, then we speculated that NNS activity might be higher during G2 when Sen1 levels rise. To test this hypothesis, we monitored termination of *SNR13*, whose termination depends on NNS, APT, and the CF1A subunit Pcf11 (Grzechnik et al., 2015, Nedea et al., 2003, Steinmetz and Brow, 2003, Steinmetz et al., 2001). When wild-type termination fails, the *SNR13* transcript is extended to the PAS of the downstream *TRS31* gene, forming a stable bi-cistronic RNA that allows quantification of transcription readthrough. Comparing *SNR13* transcripts in G1- and PM-arrested cells (Figure 1E), we observed no readthrough in a wild-type strain but saw marked differences in the sensitized background of temperature-sensitive mutations in Pcf11 or the APT component Ssu72. Both *ssu72-2* and *pcf11-9* strains (at permissive and non-permissive temperatures) show significantly less readthrough during mitotic arrest, when Sen1 protein levels are higher. A similar effect is seen in an *nrd1-102* mutant. In contrast, when Sen1 itself is compromised by the *sen1-1* mutation, termination is equally defective during G1 and PM, suggesting that limiting Sen1 in G1 causes reduced termination efficiency at *SNR13*.

In summary, we conclude that Sen1 protein levels vary throughout the cell cycle and that this variation affects transcription termination efficiency at *SNR13*.

### Sen1 Is Degraded by the Ubiquitin-Proteasome System

**Figure 2:**
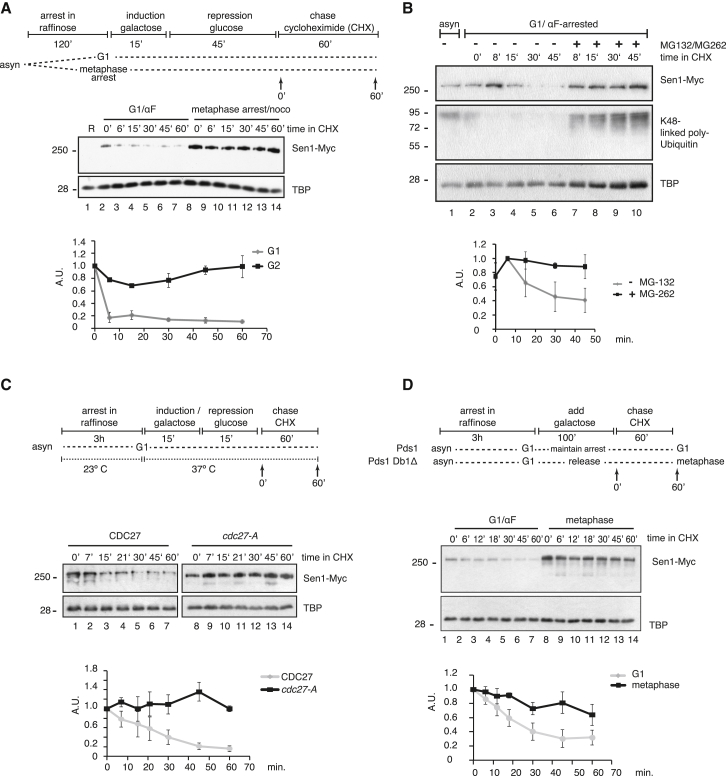
Sen1 Is Ubiquitylated and Degraded by the Proteasome (A) Sen1 stability differs in G1- and PM-arrested cells. pGSen1Myc-transformed cells (*bar1Δ*) were grown in raffinose-containing medium and arrested with 0.15 μg/mL αF or 15 μg/mL nocodazole. Sen1-Myc expression was induced by addition of 2% galactose for 15 min, followed by transcription repression by addition of 2% glucose and translation inhibition after 1 hr by addition of 1 mg/mL CHX. Whole-cell extracts from the indicated time points were assayed for Sen1-Myc levels; quantification (average of n = 3 and SEM) is graphed below. See Figure S2A for FACS analysis. (B) Sen1 degradation in G1 depends on proteasome function. Sen1-Myc cells (*bar1Δ, pdr5Δ*) were αF-arrested and treated with 1 mg/mL CHX. The culture was split in half and treated with DMSO (lanes 2–6) or 140 μM MG-132 and 20 μM MG-262 (lanes 7–10). See Figure S2B for FACS analysis. Graph: average of n = 3 with SEM. (C) Sen1 is stabilized in the APC mutant *cdc27-A*. pGSen1Myc-transformed *cdc27-A* and *CDC27* cells were αF-arrested in raffinose at 23°C and shifted to 37°C, and Sen1-Myc expression was induced with 2% galactose for 15 min, after which 2% glucose was added. CHX was added after 30 min, and residual Sen1 was analyzed as before. Graph: average of n = 3 *CDC27* and n = 4 *cdc27-A* with SEM. See Figure S2D for FACS analysis. (D) Sen1 is stabilized in metaphase-arrested cells. A galactose-inducible, non-cleavable Pds1 (Pds1Db1Δ) was integrated into Sen1-Myc cells. After αF arrest in raffinose, Pds1Db1Δ cells were released into galactose-containing medium, and CHX was added after 80 min., when most cells were arrested in metaphase (lanes 8–14). This was compared with Sen1-Myc wild-type cells, αF-arrested, and maintained in galactose for 80 min (lanes 1–7). Quantification (average and SEM, n = 4–5) is shown at the bottom. See Figure S2E for FACS analysis.

To determine whether Sen1 protein levels change through differential protein degradation, we performed a translation shutoff experiment. A plasmid-encoded, C-terminally Myc-tagged *SEN1* under control of the galactose-inducible *GAL1* promoter (pGSen1Myc) was expressed for 1 hr in G1- or PM-arrested cells prior to translation inhibition with cycloheximide (CHX) (Figure S2A). In G1-arrested cells, most Sen1 is lost 6 min after translational shutoff (Figure 2A). In contrast, higher levels of Sen1 accumulate in mitosis-arrested cells, and these remain high when CHX is added. This suggests that Sen1 is unstable during G1.

The majority of regulated protein turnover in eukaryotic cells is mediated by the ubiquitin-proteasome system (reviewed in Finley et al., 2012). To test whether the proteasome is responsible for Sen1 degradation in G1, we measured the half-life of endogenous Sen1-Myc in the presence of the proteasome inhibitors MG-132 and MG-262 (Gaczynska and Osmulski, 2005; Figures 2B and S2B). We note that, in αF-arrested cells, endogenous Sen1 is degraded with somewhat slower kinetics than the plasmid-encoded Sen1 (compare Figure 2A with Figure 2B). However, upon proteasome inhibition, polyubiquitin accumulates, and Sen1 degradation is clearly prevented.

### Sen1 Degradation Is Initiated through APC-Mediated Ubiquitylation

G1-specific degradation of proteins is often initiated through APC^Cdh1^. In the temperature-sensitive APC subunit mutant *cdc27-A*, Sen1 expressed from pGSen1Myc at non-permissive temperatures is noticeably stabilized after CHX treatment, arguing that Sen1 is an APC substrate (Figures 2C and S2D). Similarly, protein steady-state abundance increases modestly in the *cdc16-123* temperature-sensitive mutant of APC (Figure S2C). In early mitosis, APC recognizes substrates through the alternative adaptor Cdc20, and this activity initiates chromosome segregation through degradation of Pds1. Because APC is inhibited by nocodazole activation of the spindle attachment checkpoint (SAC; Sivakumar and Gorbsky, 2015), we wished to exclude that Sen1 stabilization by nocodazole was caused by APC inhibition. To this end, we arrested cells in mitosis by artificially stabilizing Pds1 to prevent chromosome segregation (Figures 2D and S2E). In the presence of active APC in mostly metaphase-arrested cells, endogenous Sen1 remains stabilized, albeit to a lesser extent than seen in APC inhibited cells (Figure 2A). We conclude that APC is responsible for Sen1 degradation during G1 and contributes to its modest turnover in early mitosis.

### Levels of Sen1 Protein Affect Viability

**Figure 3:**
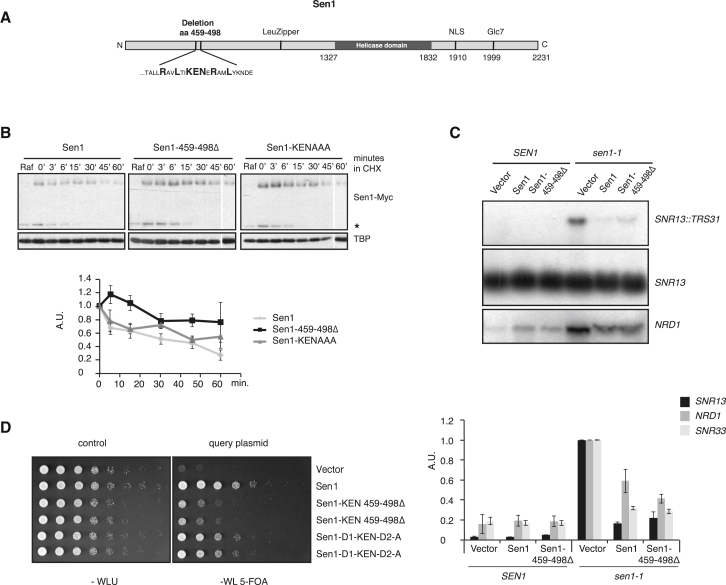
A 40-aa Region within Sen1 Contributes to Its Instability in G1 (A) Schematic model of Sen1 domain organization. LeuZipper, a putative leucine zipper; NLS, nuclear localization sequence; Glc7, Glc7 binding motif. (B) Deletion of aa 459–498 leads to Sen1 stabilization. 1 mg/mL CHX was added to αF-arrested wild-type cells (*bar1Δ)* transformed with pGSen1Myc variants Sen1, Sen-459-498Δ, or Sen1-KEN-A as described in Figure 2A. The 60-min time points in the center and at the right are from a separate gel. Bottom graph: n = 4–5, SEM. The asterisk denotes a Myc-responsive band possibly stemming from an internal promoter within Sen1, whose degradation is unchanged. (C) RNA blot analysis (1% agarose) of 15 μg RNA isolated from *SEN1* or *sen1-1* cells transformed with vector, pGSen1Myc (Sen1), or Sen1-458-498Δ and induced with galactose for 3 hr at a permissive temperature prior to a 30-min shift to a non-permissive temperature. RNA blots were probed against *SNR13* or *NRD1* (top). Bottom: quantification normalized to *sen1-1* vector readthrough (n = 3, SEM). (D) Plasmid shuffle assay to test for the ability of query constructs to support viability. A centromeric *URA3* plasmid carrying *SEN1* (pRS416 ± 700Sen1) maintains the viability of a *sen1Δ* strain. Transformation with a query plasmid (vector, Sen1, Sen1-459-498Δ, or Sen1-D1-KEN-D2-A) and selection against the *URA3* plasmid (with 5-fluorouracil [5-FOA]) leaves the query plasmid to complement the loss of *SEN1.* Five-fold serial dilutions. Selection medium: W, tryptophan; L, leucine; U, uracil.

The APC adaptors Cdc20 and Cdh1 recognize distinct amino acid (aa) motifs in their respective substrates, which aids temporal separation of substrate degradation. However, neither the destruction box (D-box) sequence (RXXLXXXXN, Cdc20) nor the lysine, glutamic acid, asparagine (KEN)-box (RxxxxxKEN, Cdh1) are unambiguously defined, and many substrates carry shortened, combined, or even alternative motifs (Sivakumar and Gorbsky, 2015). We reasoned that abrogating APC-mediated Sen1 turnover should allow us to study the biological significance of Sen1 degradation and therefore examined its aa sequence for potential minimal APC degradation motifs (RxxL and RxxxxxKEN). Although we found no APC recognition motifs within aa 552–659, deletion of which had earlier been shown to increase Sen1 levels (DeMarini et al., 1995), we did find a cluster of two potential D-boxes and a KEN box within aa 480–493 (Figures 3A and S3A). Deletion of 40 aa, including these boxes (aa 459–498), led to marked stabilization of the protein expressed from pGSen1Myc-459-498Δ in G1 (Figures 3B and S3B). However, alanine substitution of KEN within this box failed to stabilize the protein. Unfortunately, other alanine substitutions lead to protein destabilization, making it difficult to further dissect the aa requirement for Sen1 degradation within this region and test whether D-boxes contributed to Sen1 destabilization. Consequently, our analysis supports the view that Sen1 degradation depends on aa that resemble APC motifs but does not allow us to conclude unequivocally whether Sen1 acts solely as an APC^Cdh1^ substrate.

To study the phenotype of slowed Sen1 turnover, we sought to replace genomic Sen1 with the Sen1-459-498Δ allele in a plasmid shuffle assay. Because *SEN1* is essential, *sen1Δ* cells die when an empty vector is shuffled but survive when the shuffle vector carries wild-type *SEN1* (Figure 3C). Surprisingly, cells are still extremely sick when expressing only Sen1-459-498Δ protein, suggesting a correlation between Sen1 protein stabilization in G1 and reduced cell fitness. Alanine substitution of the potential degradation motifs evoked mild growth retardation, indicating that these APC-like motifs may contribute to the regulation of Sen1 (Figure S3C).

We verified that the various mutant alleles retained Sen1 function by testing their ability to complement the temperature sensitivity of the *sen1-1* mutant (Figure S3D) and found that the Sen1-459-498Δ allele was still functional as a termination factor, capable of suppressing the *sen1-1* transcription termination defect in various genomic loci (Figures 3D and S3E).

Altogether, our data suggest that Sen1 is a substrate for ubiquitin-proteasome system-mediated degradation, preferentially during G1, and that interference with this regulation reduces cell viability.

### Toxicity of Sen1 Overexpression Is Related to Its Termination Function

**Figure 4:**
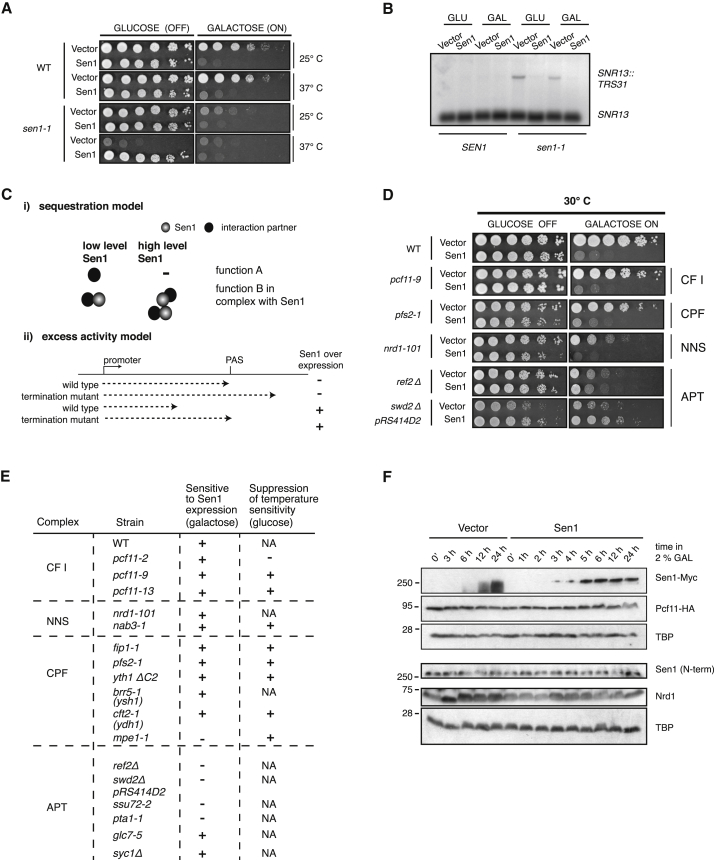
Sen1 High Copy Expression Is Toxic (A) Sen1 expression from the multi-copy pGSen1Myc plasmid in *SEN1* and *sen1-1* cells. 5-fold serial dilutions were grown on selective medium with either glucose (repressing) or galactose (inducing) as the carbon source at 25°C (permissive temperature) or 37°C (non-permissive temperature). (B) RNA blot analysis of *SNR13*. RNA was extracted from *SEN1* or *sen1-1* cells transformed with vector or pGSen1Myc. Cells were grown at 25°C in raffinose, expression was induced for 3 hr with 2% galactose or repressed with 2% glucose, and cells were shifted to a non-permissive temperature for 30 min. 20 μg RNA was separated on a 1% agarose gel, and the RNA blot was probed against *SNR13.* (Ci) Sequestration model. Sen1-interacting proteins are titrated away from other cellular functions (function A). (Cii) Excess activity model. Cells with elevated Sen1 die because transcription termination occurs prematurely. Transcription termination mutants tolerate elevated Sen1 levels by shifting the termination window back closer to the wild-type termination site. (D) Serial 5-fold dilutions of several termination factor mutants transformed with vector or pGSen1Myc. (E) Summary of phenotypes associated with Sen1 high copy expression in various termination mutants. +, yes; −, no; NA, non-applicable. (F) Immunoblot of samples taken from Pcf11-hemagglutinin (HA) wild-type cells transformed with vector or pGSen1Myc and induced for the indicated times with 2% galactose.

Given the reduced viability of *sen1-459-498Δ* cells, we sought a more amenable approach for studying the phenotype of increased Sen1 concentration in G1. Performing CHX chases in αF-arrested cells, we previously observed that prolonged expression from the multi-copy galactose-inducible pGSen1Myc led to Sen1 stabilization, perhaps by overwhelming the proteasome. To observe the long-term consequences of persistent Sen1 expression, we compared growth when expression from pGSen1Myc was induced or repressed. Although cells grew on repressive glucose, they were unable to grow on galactose medium (Figure 4A). Even in *sen1-1* cells, which die when the mutant Sen1 is destabilized at non-permissive temperatures (see Figure 4A, bottom, 37°C), expressing pGSen1Myc on galactose is toxic. In contrast, on glucose, where the *pGAL1* on pGSen1Myc is repressed, the low “leaky” expression level of wild-type Sen1 complements the *sen1-1* temperature sensitivity. Importantly, both low- and high-level expression can suppress the *sen1-1* termination defect at *SNR13* and *SNR33* (Figures 4B and S4A), suggesting that minute amounts of Sen1 are sufficient to provide a *sen1-1* strain with adequate Sen1 function but also that toxicity is not caused by a dominant-negative effect of plasmid-expressed Sen1. Overall, these results suggest that there is a window of optimal Sen1 concentration range, outside of which cells die. Taking our half-life measurements into account, we predict that sensitivity to increased Sen1 levels mainly arises during the G1 phase of the cell cycle.

In an attempt to understand the observed toxicity of Sen1 expression, we considered two different but not mutually exclusive models. According to the sequestration model (Figure 4Ci), excess Sen1 titrates some interaction partner, resulting in the partner’s insufficient activity. Alternatively, increased Sen1 activity (particularly during G1) could impair proper cellular function. For example, excessive termination might disrupt gene expression (Figure 4Cii). To test for the sequestration model, pGSen1Myc was expressed in genetic backgrounds that would abolish interaction with potential interaction partners (Figure S4B). Deletion of *RNT1* or *RAD2*, two non-essential Sen1 interaction partners, did not alleviate the toxicity of Sen1 expression (Ursic et al., 2004), nor did deletion of *SRS2*, which is synthetic lethal with *sen1-1* (Mischo et al., 2011). Similarly, point mutations in Sen1 residues that abrogate interaction with the essential proteins Rpb1 (R302W; Chinchilla et al., 2012) or Glc7 (F2003A; Nedea et al., 2008) remained toxic when expressed from galactose-inducible plasmids (Figure S4C).

To test the “excess activity model,” we reasoned that if higher cellular Sen1 levels caused excessive and cytotoxic transcription termination, then such overactivity might be offset, and therefore tolerated, in transcription termination mutants (Figure 4Cii). Accordingly, a collection of mutant strains (either deletion of non-essential genes or temperature-sensitive point mutants for essential genes) was challenged with pGSen1Myc (Figures 4D and 4E and S4D–S4G).

Three different outcomes were observed for the tested collection of mutant strains. First, as observed for *sen1-1* (Figure 4A), leaky *GAL1* promoter expression of Sen1 on glucose suppresses the temperature sensitivity of the NNS and CFI mutants *nab3-11*, *pcf11-9*, and *pcf11-13* (Figures S4D and S4E), which specifically disrupt NNS termination (Kim et al., 2006, Steinmetz et al., 2001). Nonetheless, sensitivity to *pGAL1*-induced Sen1 expression on galactose persists. Second, with other CFI, NNS, and CPF mutants, higher-level Sen1 expression is toxic (Figures 4D and 4E and S4D–S4G), and low-level Sen1 fails to suppress temperature sensitivity.

**Figure 5:**
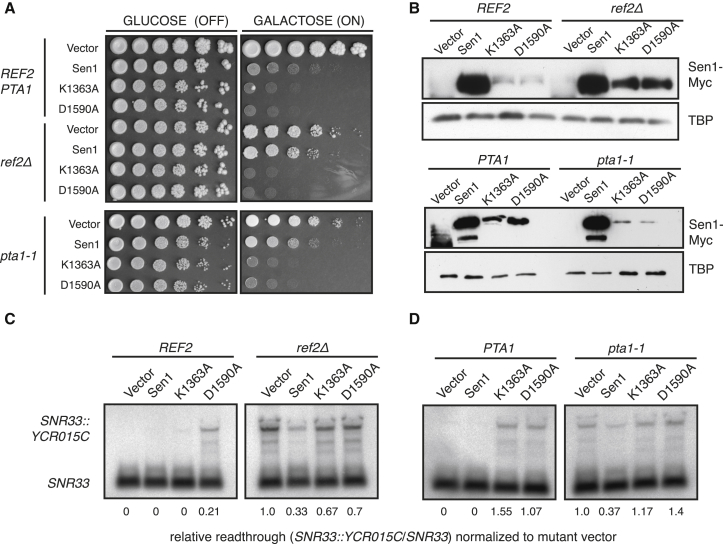
Sen1 High Copy Expression Suppresses Termination Defects in APT Mutants (A) Serial 5-fold dilutions of wild-type, *ref2Δ*, or *pta1-1* cells transformed with vector, pGSen1Myc, or two catalytically inactive point mutants of Sen1 in the Walker A and B motifs (pGSM-K1363A and pGSM-D1590A). (B) Immunoblot analysis of whole-cell extracts from cells induced with 2% galactose to express the indicated constructs. Because of different growth rates, the induction time was varied for the wild-type-*ref2Δ* (6 hr) and the wild-type-*pta1-1* (13 hr) pair. (C) RNA blot analysis of *SNR33* in *REF2* or *ref2Δ* cells. 20 μg RNA was separated on a 1% agarose gel and normalized to the *SNR33::YCR015c/SNR33* signal in the *ref2Δ* vector samples. (D) RNA blot analysis of *SNR33* in *PTA1* or *pta1-1* cells, performed as in (C).

Strikingly, several mutants in the CPF-associated APT complex withstand galactose-induced Sen1 expression (*ref2Δ*, *pta1-1*, *ssu72-2*, or *swd2Δ* [at 30°C]; Figure S4G). Although APT is critical for NNS termination, different mutations show varying substrate specificity, possibly explaining why *glc7* and *syc1* mutants are still sensitive to Sen1 induction. Also tolerating Sen1 expression was the CPF subunit *mpe1-1* (Figure S4F), which shows some NNS transcription termination defects (M. Kim, personal communication). We excluded the trivial possibility that induction of pGSen1Myc altered the expression of other termination factors by observing Nrd1 and Pcf11 levels (Figure 4F). Over 24 hr induction of pGSen1Myc, neither Pcf11 nor Nrd1 steady-state levels change. Importantly, the overall levels of Sen1 remain low, arguing that induction of Sen1-Myc is countered by lowering overall Sen1 expression. Similarly, we rejected the possibility that Sen1-Myc expression is impaired in two APT mutants that tolerate pGSen1Myc induction. Although the slow mutant growth required longer induction times, after 6 and 13 hr, respectively, Sen1 expression was equal in wild-type and *ref2Δ* or *pta1-1* mutants (Figure 5B). Therefore, from this candidate approach, we conclude that Sen1 overexpression toxicity is specifically suppressed by mutations in APT. In light of our two models, these data support the notion that Sen1 overexpression can be toxic because of increased transcription termination activity, which can be offset in cells with decreased APT (Figure 4Cii).

To provide direct evidence for this hypothesis, we tested whether Sen1 plasmid expression can suppress the accumulation of read-through *SNR33::YCR015C* RNA in APT mutants (Figures 5C and 5D). When pGSen1Myc is induced in either *ref2Δ* or *pta1-1* cells, readthrough transcription is suppressed by 60%. Suppression requires Sen1 activity because point mutants in helicase domain I (K1363A in the Walker A motif, essential for NTP binding) or helicase domain II (D1590A in the Walker B motif, essential for Mg^2+^binding) fail to alleviate the *ref2Δ* and *pta1-1* termination defects (Figures 5C and 5D). Both mutant proteins are dominant-negative, as can be seen by accumulation of readthrough transcripts in the wild-type. Furthermore, cells continually expressing these catalytically dead proteins die even when APT is mutated (Figure 5A), likely explaining their lower steady-state levels observed by immunoblot (Figure 5B).

### Sen1 Increases Termination Efficiency

A further prediction from the excess activity model is that galactose-induced Sen1 expression in wild-type cells should lead to premature termination. To identify transcripts that were affected by Sen1-Myc expression genome-wide, we employed native elongating transcript sequencing (NET-seq) (Churchman and Weissman, 2012). Because NET-seq maps nascent transcripts, its readout is independent of transcript stability and can therefore detect changes in stable and unstable ncRNA that require Sen1 for termination. We isolated duplicate samples for NET-seq in pGSen1Myc- or vector-transformed cells after 3 hr of induction, where substantial Sen1 expression was visible in the NET-seq strain but cells are still viable (Figures S5Ai–S5Aiii).

**Figure 6:**
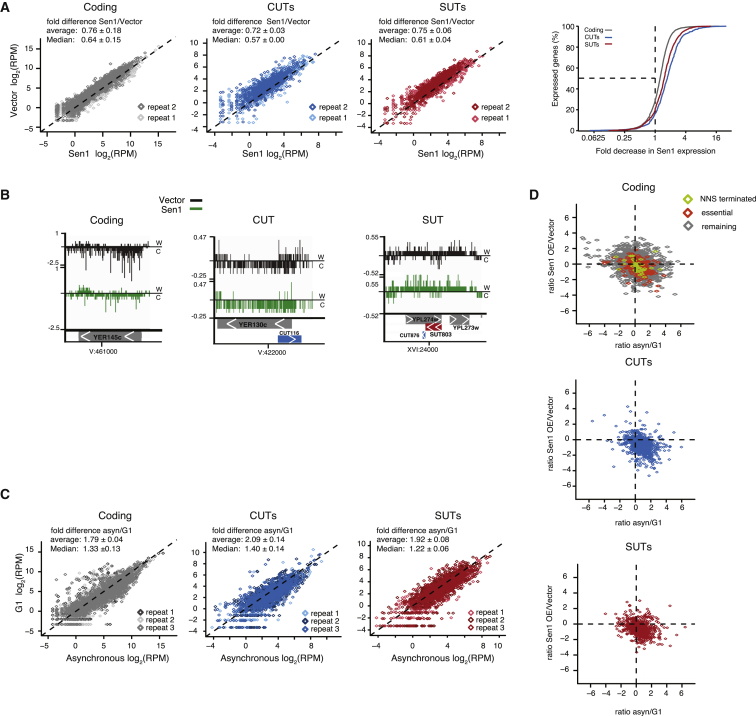
Summary of NET-Seq Results Shown is a NETseq analysis of cells that were asynchronous, αF- or nocodazole-arrested, or induced with 2% galactose for 3 hr to express vector or pGSen1Myc (Sen1). (A) NET-seq values in reads per million (RPM) of two biological repeats for coding mRNA (gray, rep1 n = 6554, rep2 n = 6601), CUTs (blue, n = 922 and 925), and SUTs (red, n = 835 and 843) in Sen1 or vector, represented as scatterplots. Right: cumulative distribution of the fold decrease in expression for coding genes, CUTs, and SUTs after Sen1 induction. (B) Genome browser view (igv; http://software.broadinstitute.org/software/igv/) for individual examples depicting lower Pol II reads in Sen1 samples: *YER145c*, CUT116, and SUT803 (based on rep1). (C) RPM values for coding genes (rep1 n = 6,539, rep2,3 n = 6,620), CUTs (n = 924), and SUTs (rep1 n = 834, rep2,3 n = 845) in G1-arrested versus asynchronous cells, depicted as scatterplot. (D) Scatterplots of fold changes comparing Sen1/vector with G1/asynchronous. Shown are coding genes (gray), CUTs (blue), and SUTs (red). Among coding genes that are lower-expressed in G1 and Sen1 cells, essential genes (p = 2.2 × 10^−16^) and NNS-terminated genes (p = 0.043, Fisher’s exact test) are significantly overrepresented (based on rep1).

Comparison of total NET-seq reads within transcription units reveals that this short Sen1 induction leads to a marked reduction of Pol II-associated transcripts at cryptic unstable transcripts (CUTs), stable unannotated transcripts (SUTs) (1.5- to 2-fold), and, to a lesser extent, at coding genes (Figure 6A). Individual snapshots of *YER145c*, *CUT116*, and *SUT803* exemplify these changes (Figure 6B). This decrease in nascent transcription could indicate that Sen1-overexpressing cells die because of an overall reduction in steady-state levels of these RNA classes. However, RNA blots for some of the most affected coding and several strongly affected essential genes showed that mRNA levels remain largely unchanged even after 24 hr of Sen1 induction (e.g., Ssu72; Figure S5B). Thus, although Sen1 expression affects nascent RNA production, mRNA steady-state levels may be less affected because of “buffering” of RNA degradation, which can obscure changes in transcription rates (Sun et al., 2013). This result importantly suggests that reduced mRNA levels are unlikely to cause Sen1 toxicity.

Given the fluctuations in Sen1 levels over the cell cycle, we asked whether genes whose expression changes during the cell cycle correlate with genes affected by Sen1 overexpression. Therefore, NET-seq was performed on asynchronous, G1- and PM-arrested cells. Surprisingly, mean NET-seq signals are reduced almost 2-fold in G1-arrested cells compared with asynchronous or mitosis-arrested cells (Figures 6C and S5Ci–S5Ciii). In fact, many transcripts reduced in G1 are also reduced by Sen1 overexpression, with essential and NNS-terminated genes being significantly overrepresented (Figure 6D). However, RNA blot analysis of RNA isolated from G1 arrested cells overexpressing Sen1 again failed to show effects on the steady-state level of several essential or NNS attenuated genes (data not shown). These results indicate that increased Sen1 levels during S/G2 do not lead to an overall reduction in nascent transcripts or mRNA levels.

**Figure 7:**
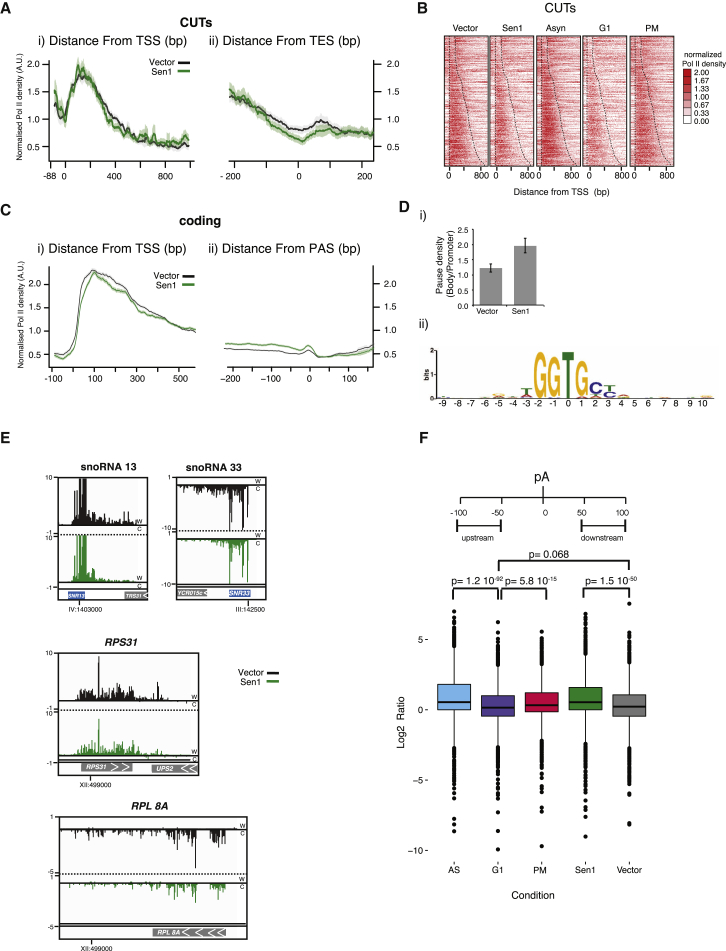
Sen1 Affects Pol II Pausing and Termination Position (A) Aggregate plot of all CUTs with a reads per kilobase of transcript per million mapped reads (RPKM) > 10 (n = 925) anchored at the transcription start site (TSS, i) or the annotated transcription end site (TES, ii). The shadow denotes a 95% confidence interval. (B) Heatmap of NET-seq reads (RPM) for all CUTs in vector, Sen1 (pGSen1Myc), asynchronous, and G1-arrested cells. (C) Aggregate plot of all coding genes with an RPKM > 10 and more than 1,000 nt (n = 2792), anchored at the TSS (i) or the poly(A) site (ii); vector (black), Sen1 (green). (Di) Ratio of Pol II pause intensity on gene bodies compared with promoter-proximal 300 bp in vector and Sen1 cells. Also see STAR Methods. (Dii) 15 nt up and downstream of non-overlapping pause sites were searched for motif enrichment using multiple expectation maximization for motif elicitation (MEME) (Bailey et al., 2009). The identified motif occurred in both samples (vector: 563 of 1,077 pause sites, p = 2.1 × 10^−639^; Sen1: 708 of 1,237 identified pause sites, p = 2.7 × 10^−672^). (E) Individual gene examples from vector and Sen1 samples showing *SNR33*, *SNR13*, *RPS31*, and *RPL8A*. The RPM scale in the igv plot is indicated. (F) Boxplot for the termination ratio of the indicated samples. The termination ratio is determined by taking the ratio of reads from 100–50 nt upstream and 50–100 nt downstream of the TES site. 5,702 coding genes that show no overlap with other transcripts 100 nt downstream of the transcript isoform-sequencing (TIF)-seq end were included in the analysis. The p values represent Student’s t test.

To deepen our analysis, we analyzed the distribution of NET-seq reads across genes to see how Sen1 overexpression affects transcriptional elongation and termination. Aggregate plots of reads along transcription units, normalized to Pol II levels, allow comparison of profile changes between different samples. At CUTs, which are terminated by the NNS pathway, Sen1 overexpression reduced transcribing Pol II around the 3′ end of the transcription unit (Figure 7Aii). This result suggests that NNS termination becomes more efficient with increased Sen1, supporting our conclusion that Sen1 can be the limiting factor in this pathway (Figures 4 and S4). Heatmaps of normalized Pol II density for individual CUTs (Figure 7B) show both a reduction of overall Pol II density and a specific reduction at CUT 3′ ends when Sen1 is plasmid-expressed. In contrast, aggregate plots and heatmaps of SUTs show a more homogeneous picture (Figures S6A and S6B). In agreement with the total read analysis, CUTs and SUTs in G1-arrested cells show an overall Pol II signal reduction but no distribution changes compared with asynchronous or mitosis-arrested cells (Figures 7B and S6B).

Finally, aggregate profiles of coding genes differ markedly between samples (Figure 7C). First, within the gene body, pGSen1Myc cells accumulate more Pol II in 3′ regions upstream of the PAS (Figure 7Cii) and relatively fewer signals around the TSS (Figure 7Ci). A moving average analysis that identifies pause sites in individual genes shows that pGSen1Myc samples are more likely to pause further downstream within the gene body (Figure 7Di). Half of the pause positions in both vector and pGSen1Myc cells carry the motif GGTG (with T being the 3′ end of the RNA; Figure 7Dii). Pol II pausing can occur transiently during transcription or indicate a Pol II molecule in the process of termination (Hyman and Moore, 1993, Larson et al., 2011, Park et al., 2004). To test whether pausing was associated with NNS termination, we examined RNA outside of the Pol II footprint for Nrd1 and Nab3 motifs. However, motif search and motif enrichment tools fail to identify Nrd1 or Nab3 motifs within 40 nt upstream of the pause site (Figure S7C; Bailey et al., 2009, Carroll et al., 2004, Carroll et al., 2007, Creamer et al., 2011). Thus, under our experimental conditions, Pol II has a propensity to pause at the sequence GGTG, with no correlation to Nrd1-Nab3 sites further upstream.

The second effect of pGSen1Myc expression apparent in the coding gene aggregate analysis is a drop in Pol II density after the PAS (Figure 7Cii), suggestive of globally increased termination efficiency. To test the termination efficiency of individual genes, a termination ratio was calculated as the ratio of reads 50–100 nt upstream divided by 100–50 nt downstream of the PAS. Genes with overlapping transcription units 100 nt downstream of the PAS on the same strand were excluded from this analysis. Sen1 overexpression clearly increases termination efficiency (average termination coefficient = 2.545 compared with Vector = 1.935). Individual traces of highly expressed genes, like small nucleolar RNAs (snoRNAs) (NNS pathway) and ribosomal genes (poly(A) pathway and failsafe), exemplify increased termination efficiency (Figure 7E).

Similar to Sen1-overexpressing cells, asynchronous or nocodazole-arrested cells have significantly higher termination coefficients than G1-arrested cells (Figure 7F). Thus, the higher levels of Sen1 in G2/M (asynchronous and mitosis-arrested cells) may induce more efficient termination.

In summary, NET-seq analysis shows that Sen1 overexpression distorts the transcription levels of CUTs and SUTs and increases the termination efficiency of CUTs and mRNA. Increased termination efficiency is also observed outside of G1, which may be correlated with higher Sen1 protein levels. Overall, this provides a biological rationale for keeping Sen1 levels low because excess Sen1 acts to trigger inappropriate or premature termination.

## Discussion

We show in this study that Sen1 protein levels are regulated through the cell cycle. Ubiquitin-proteasome system-mediated degradation decreases protein levels 3- to 10-fold in G1 relative to other cell cycle stages (Figures 1, 2, and 3). Limiting Sen1 levels appears to be essential to the cell because manipulation of this regulation through overexpression or deletion of Sen1 degradation sequences results in greatly reduced cell viability. Notably, increased levels of Sen1 have direct consequences for general Pol II occupancy and termination efficiency/position, as shown by NET-seq (Figures 6 and 7) and RNA steady state analysis (Figure 1E). Genetic experiments indicate that Sen1 toxicity results from excess termination activity (Figures 4 and 5).

Sen1, Nrd1, and Nab3 are required for termination of many common ncRNA or attenuated mRNA transcripts, genetically justifying the model of an NNS complex. Biochemical studies suggest that the whole NNS termination complex includes Pol II, cap binding complex, Rnt1 (RNase III), the exosome, and the Trf4/Air2/Mtr4 polyadenylation (TRAMP) complex (Vasiljeva and Buratowski, 2006). However, average cellular Sen1 levels are notably lower than those of Nrd1 and Nab3, suggesting that Sen1 could be rate-limiting in the NNS pathway. Although they do not co-purify with NNS, NNS-mediated termination also requires Pcf11 and APT, both components of the CPF/CF mRNA termination complex. Importantly, Sen1 has been shown to terminate Pol II *in vitro* without additional factors (Porrua and Libri, 2013). Therefore, it remains unclear whether all of these components act during every NNS termination event or whether different subsets of factors can be combined opportunistically to carry out the mechanistic steps needed to ensure efficient Pol II termination.

We sought to determine whether the abundance of Sen1 regulates NNS efficiency or might instead affect NNS-independent functions of Sen1. Our data support both possibilities. NNS termination is more efficient when Sen1 is more abundant; overall transcription at NNS-terminated CUTs is strongly affected, and a subset of mutants that reduce NNS termination suppresses Sen1 toxicity. On the other hand, 30% of mRNA-encoding genes are terminated more than 2-fold more efficiently when Sen1 concentration is high. These do not contain known Nrd1/Nab3 binding sites, nor do they belong to a particular function or pathway (gene ontology [GO] analysis). Similarly, genes that show increased occurrence of pause sites in their body do not classify into any GO term. Consequently, the lethality of increased Sen1 levels cannot be definitively connected to any particular RNA but may result from cumulative effects on many essential mRNAs as well as the overall reduction in ncRNAs (Figures 6D and 7D).

For both Sen1 (NNS)- and Rat1 (PAS)-mediated termination mechanisms, pausing of Pol II can promote termination, presumably by providing time for the “displacing” enzyme to track along the RNA and catch the elongation complex (Mischo and Proudfoot, 2013). Thus, Rpb1 mutants with slowed elongation or conditions that increase Pol II pausing partially suppress the termination and growth defects of hypomorphic *sen1* mutants (Hazelbaker et al., 2013). We therefore propose that Sen1 is recruited to and acts on paused Pol II. At many sites, recruitment occurs via Nrd1/Nab3 binding to nascent RNA and the Pol II CTD (Chinchilla et al., 2012, Conrad et al., 2000). But if RNA is accessible, then Sen1 may also terminate Pol II paused by other protein-DNA roadblocks, damaged DNA, or intrinsic DNA sequences. In view of the toxicity of Sen1 overexpression, it is conceivable that its access to paused Pol II has to be kept in check by regulating its activity or reducing the available amounts of protein. This would explain why Sen1 levels have to be higher during G2, where two sister chromatids are present and general transcriptional activity may be higher (Figure 6). The human Sen1 homolog Senataxin does not change in concentration throughout the cell cycle but alters in cellular localization, possibly also regulating its site of action in a cell cycle-dependent fashion (Yüce and West, 2013).

Finally, Sen1, but not Nrd1 and Nab3, is required to prevent collisions between replication forks and transcribing polymerases (Alzu et al., 2012). This observation further suggests that Sen1 can act independently of NNS and could explain why Sen1 is required to maintain genome stability; at paused Pol II, the dwell time of RNA at the site of negatively supercoiled DNA upstream of Pol II is higher, increasing the probability of forming R-loops. Thus, R-loop removal may be a side effect of Sen1 termination activity.

Future studies will aim to further dissect the action of Sen1 in different phases of the cell cycle. Moreover, given the drastic effects of Sen1 expression on Pol II chromatin occupancy, it is conceivable that other environmental stimuli may control Sen1 abundance. NNS action is coupled to the nutritional state of cells (Darby et al., 2012), and together with the control of Sen1 action described in this work, opens the fascinating possibility of adjusting transcription termination or, more generally, ncRNA abundance to environmental cues and stimuli.

## STAR★Methods

### Key Resources Table

**Table:** 

REAGENT or RESOURCE	SOURCE	IDENTIFIER
**Antibodies**		

anti-Myc (9E11)	House production LRI	9E11
anti-Sen1 - (raised against N-terminal stretch of Sen1)	This study	
anti-TBP	Buratowsi and Zhou (1992)	
anti-Nrd1	Steinmetz and Brow, (1998)	
anti-Nab3 (2F12)	Wilson et al., (1994); kind gift from Jeff Corden	2F12
anti-Sic1	kind gift from John Diffley	
Anti-FLAG M2 affinity gel	Sigma-Aldrich	Cat# A2220 RRID: AB10063035
Chemicals, Peptides, and Recombinant Proteins		
Alpha-Factor	House production LRI	
3x FLAG Peptide	Sigma-Aldrich	Cat#F4799
Cycloheximide	Sigma-Aldrich	C7698
Nocodazole	Sigma-Aldrich	M1404
MG-132	MERCK	474790
MG-262	Stratech	A8179-APE

**Deposited Data**		

Raw data deposition at Mendeley	This study	Mendeley: https://doi.org/10.17632/bsrvhwgs5j.1
Raw Sequencing data	This study	GEO: GSE86419

**Experimental Models: Organisms/Strains**		

BMA64 (MATa *ura3-1 Δtrp1 ade2-1 leu2-3,112 his3-11,15)*	Chanfreau et al., (1998)	(YF1342)
Brr5-1 (YSN399; MATα *his3Δ200 leu2Δ1 ura3-52 brr5-1ade2-100 lys2-801* (amber))	Noble and Guthrie, (1996)	(HY431/YF1437)
BY4741 (MATa *his3Δ1 leu2Δ0 ura3Δ0 met15Δ0)*	Euroscarf	(FY44)
BYSHM (MATa *his3Δ1 leu2Δ0 ura3Δ0 met15Δ0 SEN1::His^∗^6-TEV-Myc^∗^18::URA3)*	This study	(HY202)
BYSHM Pdr5D (MATa *his3Δ1 leu2Δ0 ura3Δ0 met15Δ*0 *SEN1*::*His^∗^6-TEV-Myc^∗^18::URA3 pdr5Δ::KanMX)*	This study	(HY270)
cdc16-123 ((W303) MATa *his3-11,15 leu2-3,112 trp1-1 ura3-1 can1-100 cdc16-123)*	L. Drury/J. Diffley	(FY59)
cdc27-A (*MATa bar1::hisG, cdc27-A, ura3, leu2, trp1, his3, ade2* (backcrossed to W303 four times))	A. Amon	(HY500/ YF2412)
Cft2-1/Ydh1Δ (MATa *his3Δ1 leu2Δ0 ura3Δ0 met15Δ0 ydh1Δ::KanMX* [pAK21 = ydh1-1 LEU2 CEN])	Kyburz et al., (2003)	(HY403/YF2367)
Fip1-1 (LM94; MATα *leu2-3,112 trp1- ura3-52 his4- fip1Δ::LEU2* [pIA23 = fip1-1 (L99F Q216Stop) TRP1 CEN])	Preker et al., (1995)	(HY397/YF2360)
Glc7-5 (MATa *his3-11,15 leu2-3,112 ura3-1 ade2-1 can1-100 ssd1-d2 glc7Δ::LEU2 trp1::glc7-5::TRP1)*	Andrews and Stark, (2000)	(HY406/YF2369)
Mpe1-1 ((W303-1B) MATα *his3-11,15 leu2-3,112 trp1-1 ura3-1 ade2-1 mpe1-1 (F9S, Q268K, K337F, K354STOP))*	Vo et al., (2001)	(HY430/YF1982)
Nab3-11 (YPN103; (W303-1B) MATα *his3-11,15 leu2-3,112 trp1-1 ura3-1 ade2- can1-100 nab3-11)*	Conrad et al., (2000)	(HY371/YF1471)
nrd1-101 (YJC1282; BY4741 (S288C) MATa *his3Δ1 leu2Δ0 ura3Δ0 met15Δ0 nrd1-101::HA)*	Jeff Corden	(HY127/YF2347)
nrd1-102 ([nrd1 (V379G)]; (S288C) MATa *leu2Δ1 trp1Δ63 ura3-52 nrd1-102 [nrd1 (V379G)])*	Minkyu Kim	(HY479/YSB2079)
Pcf11-13 ((W303, *RAD5+*) MATa *his3-11,15 leu2-3,112 trp1-1 ura3-1 ade2-1 can1-100 pcf11Δ::TRP1* [pNOPL-pcf11-13 (pcf11-13 (D68A,S69A,I170A), LEU2 CEN/ARS)])	This study	(HY312)
Pcf11-2 (NA65; (W303-1B) MATa *his3-11,15 leu2-3,112 trp1Δ ura3-1 ade2-1 pcf11-2 (E232G, D280G, C424R, S538G, F562S, S579P))*	Amrani et al., (1997)	(HY366/YF1434)
Pcf11-9 (NA67; (W303-1B) MATa *his3-11,15 leu2-3,112 trp1Δ ura3-1 ade2-1 pcf11-9 (A66D, S190P, R198G, R227G, E354V, K435V))*	Amrani et al., (1997)	(HY305/YF1435)
PFS2 (MO12; (W303-1B) *his3-11,15 leu2-3,112 trp1Δ ura3-1 ade2-1 pfs2Δ::TRP1* [pFL36-PFS2 = PFS2 LEU2 CEN])	Ohnacker et al., (2000)	(HY407/YF2370)
Pfs2-1 (MO17; (W303-1B) *his3-11,15 leu2-3,112 trp1Δ ura3-1 ade2-1 pfs2Δ::TRP1* [pFL36-pfs2-1 = pfs2-1 LEU2 CEN])	Ohnacker et al., (2000)	(HY408/YF2371)
Pta1-1 (P0C8-23d; MATa *leu2Δ1 trp1Δ101 ura3-52 pta1-1 ade2-1 lys2-)*	O’Connor and Peebles, (1992)	(HY379/YF175)
Rad2Δ ((S288C) MATa *his3Δ1 leu2Δ0 ura3Δ0 met15Δ0 rad2Δ::KanMX)*	Winzeler et al., (1999)	(YF2230)
Ref2Δ ((S288C) MATa *his3Δ1 leu2Δ0 ura3Δ0 met15Δ0 ref2Δ::KanMX)*	Winzeler et al., (1999)	(HY361/YF1996)
rnt1Δ W303) (MATa *his3-11,15 leu2-3,112 Δtrp1 ura3-1 ade2-1 rnt1Δ::HIS3)*	Chanfreau et al., (1998)	(HY163/YF1343)
Shuffle strain ((BY4743; S288C) *ura3Δ0 leu2Δ0 trp1Δ::LEU2/Kan*^*R*^*his3Δ1 met15Δ0 sen1Δ::KanMX* [pRS416 +-700 Sen1])	This study	(HY459/YSB3181)
Srs2Δ (BY4741 (S288C) MATa *his3Δ1 leu2Δ0 ura3Δ0 met15Δ0 srs2Δ::KanMX)*	Winzeler et al., (1999)	YF2355
Ssu72-2 (MATa *ura3-52 leu2-3,112 his3Δ200 ssu72-2 (R129A))*	Pappas and Hampsey (2000)	(HY378/YF1374)
Swd2Δ pRS414 D2 (MATa *ura3Δ0 leu2Δ0 his3Δ1 met15Δ0 swd2Δ::KanMX* [pRS414 +-700 Sen1 D2])	This study	(HY446)
Syc1Δ ((S288C) MATa *his3Δ1 leu2Δ0 ura3Δ0 met15Δ0 syc1Δ::KanMX)*	Winzeler et al., (1999)	(HY365/ YF2354)
W303 RAD5+ ((W303) MATa *his3-11,15 leu2-3,112 trp1-1 ura3-1 ade2-1 can1-100)*	Andres Aguilera	(HY307/YF2329)
W303-1A (MATa *his3-11,15 leu2-3,112 trp1-1 ura3-52 can1-100)*	Andres Aguilera	(FY1)
W303bar1Δ ((W303) MATa *his3-11,15 leu2-3,112 trp1-1 ura3-52 ade2-1 can1-100 bar1::HYG)*	Mischo et al., (2011)	(HY115/YF2348)
WF1ASHM (MATa *leu2-3,112 trp1-1 ura3-1 SEN1::His6::TEV::Myc9::TRP1)*	This study	(HY185)
WF1ASHM Pds1DbΔ (WF1ASHM with p258 (Pds1Δdb) integrated.)	This study	(HY509)
WF1B (Matα *ura3-1 leu2-3,112 trp1-1 his3-11,15 can1-100 sen1-1)*	Mischo et al., (2011)	(HY71/YF2349)
WF1D (MATa *ade2 his3-11,15 leu2-3,112 trp1-1 ura3 sen1-1)*	Mischo et al., (2011)	(HY73)
YTH1 (YT2; MATa *his3- leu2- trp1Δ ura3- ade2- yth1Δ::TRP1* [YCplac11-YTH1 = YTH1 LEU2 CEN])	Tacahashi et al., (2003)	(HY400/YF2364)
Yth1 DC2 (YT5; MATa *his3- leu2- trp1Δ ura3- ade2- yth1Δ::TRP1* [YCplac11-yth1ΔC2 = yth1ΔC2 (amino acids 1-147 present) LEU2 CEN])	Tacahashi et al., (2003)	(HY402/YF2366)

**Oligonucleotides**		

sCR1 up (GGCTGTAATGGCTTTCTGGTG)	This study	N/A
sCR1 dw (CACAATGTGCGAGTAAATCCTG)	This study	N/A
hm331 Sen1 Pst1 3′ 6369 (CATCATCTGCAGCTCGAAGAACCAC CGGATAAAAC)	This study	N/A
SB#1309 SNR13-60 (TTATAAATGGCATCTCAAATCGTC)	This study	N/A
SB#1310 SNR13+124 to end (GGTCAGATAAAAGTAAAAAAA GGTAGC)	This study	N/A
hm427 SNR 13 3′ SacII MboI (GTCACCGCGGGATCGGATGGT GATAGTACTCCCTGTC)	This study	N/A
SB#1319 snr33 Pro-up (CGGAACGGTACATAAGAATAGAAGAG)	This study	N/A
SB#1322 snR33 3UTR low +288 (TAAAGAAAACGATAAGA ACTAACC)	This study	N/A
hm527 Sen1Opt F2003A F (GGTAAGAAAAAGAACAACAAGCA CGTGTGCGCCTCCGATGATGTTTCTTCATTCC)	This study	N/A
hm529 Sen1Opt R302W F (CGTTGTTTCTCAATTCTGGTCTTG GTTATTGCCAGTTTTCAAC)	This study	N/A
hm510 Sopt K1363A R (CGATAATACCCAAAATAGTCTTAGTG GCGCCAGTACCTGGTGGACCTTG)	This study	N/A
hm514 Sopt D1590A R (CGGTGCATTGACAAGCTTCAGCGAT AATAACGGTATCGAAC)	This study	N/A
hm472 S D1 3′ (CATTTATAATAAACAGATGCGC)	This study	N/A
hm492 Sopt D4 R (GGCAATAATTCTCAAGAAAGCCATG)	This study	N/A
hm493 Sopt D5 F 1494 (TTCGAAACTGTCTTGTTGACCAAGAC)	This study	N/A
hm480 D4 R 1374 IIA (GGCTATTATACGCAGGAACGCC)	This study	N/A
hm477 S D5 F 1494 (TTTGAAACAGTACTGTTGACTAAAAC)	This study	N/A
hm539 F2003A introd Pml1 (GGTAAAAAGAAAAATAACAAACA CGTGTGTGCTTCGGATGATGTTAGTTTCATACC)	This study	N/A
hm473 S D2 F 2067 (AATACATTTGACGTTGAGGGTAGAC)	This study	N/A
hm550 ssu72 5′ 53+ (CAACAATCGTTCAATGGAATCGC)	This study	N/A
hm551 ssu72 3′ 311 (CTTTCTTGCCATTTTTCAGGTGC)	This study	N/A
SB#1623 Nrd1 ORF0-down (CTTATGTTCAAGTTTAAAGGAGGAC)	This study	N/A
SB#1640 Nrd1 1aa-up(+1) (ATGCAGCAGGACGACGATTTTCAA)	This study	N/A
SB#3861 SEN1 - Dbox1mut R (AGTCGCCACAGCTGCCAACAAG GCAGTTGCTGAATT)	This study	N/A
SB#3856 2 SEN1 - KEN WT F (ATTAAGGAAAATGAAAGGGCAA TGCTTTATAAGAATGATG)	This study	N/A
SB#3860 3 SEN1 - Dbox1wt R (AGTCAACACAGCCCTCAACAA GGCAGTTGCTGAATT)	This study	N/A
SB#3859 4 SEN1 - KEN-AAA+Dbox2mut F (ATTGCAGCGGCCG AAGCGGCAATGGCGTATAAGAATGATGAATTTGAA)	This study	N/A
SB#3858 5 SEN1 - Dbox2mut F (ATTAAGGAAAATGAAGCGGC AATGGCGTATAAGAATGATGAATTTGAA)	This study	N/A
SB#3857 6 SEN1 - KEN-AAA F (ATTGCAGCGGCCGAAAGGGC AATGCTTTATAAGAATGATGAATTTG)	This study	N/A

**Recombinant DNA (see also Table S1)**		

pYMHHM	Genescript	
pGSen1Myc	Geneart	
pGSM-F2003A	This study	
pGSM-R302W	This study	
pGSM-K1363A	This study	
pGSM-D1590A	This study	
pGSen1Myc-459-498Δ	This study	
pRS416 +-700 Sen1	This study	
pRS414 +-700 Sen1	This study	
pRS414 +-700 Sen1 459-498Δ	This study	
pRS414 +-700 Sen1 D2	This study	
pRS414 +-700 Sen1-KEN A	This study	
pRS414+-700 Sen1-Dbox2 A	This study	
pRS414+-700 Sen1-Dbox1 Dbox2 A	This study	
pRS414+-700 Sen1-Dbox1 KEN A	This study	
pRS414+-700 Sen1-KEN Dbox 2 A	This study	
pRS414+-700 Sen1-Dbox1 KEN Dbox 2 A	This study	
pRS414+-700 Sen1-Δ485-505	This study	
P258	This study	

**Software and Algorithms**		

TopHat2	Kim et al. (2013)	
HTSeq package	Anders et al., (2015)	
deepTools	Ramírez et al. (2014)	
FIMO	Grant et al. (2011)	

### Contact for Reagent and Resource Sharing

Further information and requests for resources and reagents should be directed to and will be fulfilled by the Lead Contact, Hannah Mischo (Hannah.Mischo@path.ox.ac.uk)

### Experimental model and subject details

#### Strains

Strains are listed in the Key Resources Table and were derivatives of either W303 (*MATa his3-11,15 leu2-3,112* *Δtrp1 ura3-1 ade2-1 can1-100*) or BY4741 (*MATa his3Δ1 leu2Δ0 ura3Δ0 met15Δ0*).

#### Cell growth and arrest

Cells were cultivated in YPD (10% yeast extract, 20% bacterial peptone, 2% glucose) or minimal media as indicated in the text. Unless otherwise noted, cells were cultivated at 30°C, or at 25°C for temperature sensitive strains. Prior to galactose induction, cells were grown in 2% raffinose in minimal selective media, to which 2% galactose was added for indicated times.

#### Elutriation

3x10^10^ cells were condensed from 2 L to 40 mL in media without carbon source and sonicated for 10 s. at 30%. Cells were loaded into the elutriation chamber and separated at 2700 to 1400 rpm.

#### αFactor arrest and release

Cells were grown to a density of 1.5 −2.5x 10^7^ cells/ml in minimal complete media or YPD, pH adjusted to 3.9 with HCl and cells arrested by two additions of 5 μg/ml αF at 0 and 60 min. Arrest was confirmed by microscopic observation after 90 to 120 min. Release from arrest was achieved by two washes with medium.

#### αF/HU/Nocodazole arrest

*BAR1* deleted strains were arrested at 0.5 −1x 10^7^ cells/ml without acidification by addition of 0.15 μg/ml αF for 90-120 min. *BAR1* carrying strains were arrested after acidification of the media to pH 3.9 with twice 5 μg/ml αF (60 and 60 min., 120 min total).

Cells were arrested at densities of 1x 10^7^ cells in S-phase with hydroxyurea (SIGMA, H8627) added to 0.2 M or in G2/M with 15 μg/ml nocodazole (SIGMA, M1404) in DMSO for 90 min to 2hrs, as judged by eye and depending on the growth rate of the strain. Arrest at 25°C for temperature sensitive strains was usually achieved after 3-3.5 hr.

#### Plasmid shuffle experiment

HY459 (shuffle strain sen1Δ, carrying *trp1::LEU2* and pRS416+-700Sen1) is transformed with centromeric plasmids pRS414, pRS414 +-700 Sen1 and its derivatives (see Key Resources Table). Transformants are selected on –WLU plates to select for query plasmid (-W), pRS416 +-700 Sen1 wild-type plasmid (-U) and LEU (-L), to ensure that the *trp1::LEU2* disruption is not popped out. After growth for 20 hr in liquid media, cells are spotted as five-fold serial dilutions onto –WLU plates to monitor general growth and 5-FOA containing plates to shuffle out the wild-type plasmid pRS416 +-700Sen1 and leave the query plasmid as only copy.

#### Spotting experiments

Overnight cultures are diluted to 4x 10^7^ cells/ml (Figures 4, 5, S3C, and S3D) or 0.5x 10^7^ cells/ml (Figure 4A) and spotted as 3 μl spots in 1:5 serial dilutions. Temperature sensitive strains were grown at 25° (permissive), 30° (semi-permissive) or 37°C (non-permissive temperature) as indicated in the figures.

#### Fluorescent activated cell sorting (FACS)

0.6x 10^7^ cells were fixed in 70% ethanol at −20°C until further processing. Ethanol was removed and RNA digested with 20 μg/ml RNase A in 50 mM Tris pH 8.0 for 90 min at 37°C. RNase A was removed and cells resuspended in 1 mg/ml Pepsin 0.5 M HCl in dH2O to digest cell walls for 30 min at 37°C. Cells were pelleted and then resuspended in 50 mM Tris pH 8.0. Typically 6x 10^5^ cells were stained with 0.5 μM Sytox® green (Molecular Probes S7020), briefly sonicated and analyzed in a FACS Calibur (BD).

### Method Details

#### Protein analysis

##### Whole cell extract analysis

NaOH lysis 7x 10^7^ cells are pelleted, washed in water, lysed in 100 mM NaOH for 3 min. at room temperature (RT), cooled on ice for 30 s, and spun for 5′ at 13000 rpm for 5 min. Protein pellets are resuspended in 50 μl SDS loading dye (0.06 M Tris pH 6.8, 5% glycerol, 2% SDS, 4% βME, 0.0025% BPB) and ca 0.75x 10^7^ cells loaded per lane (Kushnirov, 2000).

##### TCA whole cell extract

Washed cells were resuspended in 10% TCA, combined with an equal volume of glass beads, and broken in a MagNA-lyser (ROCHE) at 6000 rpm for 15 s. Beads were washed with 500-1000 μl 10% TCA and spun for 5 min at 9000 rpm. Air-dried protein pellets were resuspended in 50-120 μl TCA loading dye (1x SDS Laemmli dye, 0.4M Tris pH 11).

##### Cycloheximide (CHX) shutoff for plasmid expressed Sen1

pGSen1Myc transformed cells were grown in selective media with 2% raffinose to a density of 3x 10^7^ cells/ml and then arrested with αFactor or nocodazole. Upon arrest, tagged Sen1 expression was induced with 2% galactose for 15 min, then further transcription was repressed with 2% glucose for 45 min, and the chase started 60 min after galactose induction by addition of 1 mg/ml cycloheximide (50 mg/ml in DMSO) to the medium. 5 mL time points were spun, washed, flash frozen in liquid nitrogen and processed to extract using TCA. Approximately 2x10^7^ cells/lane were loaded onto a two-percentage (15 /11%)-SDS-polyacrylamide gel.

pGSen1Myc transformed cdc27-A or wild-type cells (both *bar1Δ*), were grown in selective media with 2% raffinose to a density of 0.5x 10^7^ cells/ml at 23°C and arrested with αF (0.15 μg/ml). Upon arrest cells were shifted to 37°C and concomitantly pGSen1Myc expression induced by addition of 2% galactose for 15 min. Transcription was then repressed with 2% glucose while keeping cells at 37°C for a further 15 min. A 6 mL aliquot of cells was taken after a total of 30 min. at 37°C for time 0 and immediately 1 mg/ml CHX added. All further time points were taken from cells maintained at 37°C as 6 mL aliquots. Each aliquot was processed and analyzed as indicated above.

##### CHX shutoff of endogenous Sen1

SEN1-Myc cells at 0.7x10^7^ cells/ml were arrested with 5 μg/ml αF, and upon arrest split in half. Deletion of the general drug exporter *PDR5* makes this strain sensitive to the uptake of both inhibitors (Golin et al., 2007). Both inoccules were exposed to 1 mg/ml CHX and in addition, one received 0.57% DMSO, the other 140 μM MG-132 (MERCK) and 20 μM MG-262 (Stratech). Approximately 0.3x10^7^ cells were loaded in each lane and separated on a 15/11% SDS-PAGE gel.

SEN1-Myc or SEN1-Myc Pds1Db1Δ cells were grown in raffinose to a density of 0.6x10^7^ cells/ml, arrested with 5 μg/ml αF and either (SEN1-Myc Pds1Db1Δ), washed twice with YP, resuspended to a density of 0.3x10^7^ cells/ml in the presence of 2% galactose and 50 μg/ml Pronase. Cells typically released and arrested in metaphase after 80min., at which point, the culture was condensed back to 0.6x10^7^ cells/ml and time point 0 (6 ml) was taken. 1 mg/ml CHX was added to the remaining cultures and 6 mL time points taken at indicated time points. αF arrested SEN1-Myc cells were incubated with additional αF and maintained in 2% galactose for 80’ before they were treated with CHX in parallel with the metaphase arrested culture.

#### RNA analysis

RNA was extracted from typically 6x 10^8^ cells/ml by addition of 400 μL AE buffer (50 mM sodium acetate pH 5.0, 10 mM EDTA pH 8.0), 50 μl 10% SDS and 500 μl phenol:chloroform:isoamylalcohol (PCA, 25:24:1, pH4.5) for a period of 5 min at 65°C. The aqueous phase was extracted twice with PCA and ethanol precipitated.

##### RNA Blot

RNA was separated on 1% agarose gels in MOPS and transferred by capillary force in 20 x SSC. Probes were generated by strand specific PCR with primers indicated using 32Pα-dATP and hybridized in phosphate hybridization buffer at 65°C (0.3 M phosphate buffer pH 7, 7% SDS, 0.01 g/ml BSA and 1 mM EDTA pH 8.0) and probes washed from membranes with 2 x SSC (150 mM NaCl, 15 mM sodium citrate), 0.1% SDS. Probes were generated by strand-specific labeling of a PCR product (primer pairs in brackets, 30 cycles) with the antisense (reverse) primer to generate a single stranded, internally labeled probe (one NTP replaced with labeled NTP, 30 cycles): NRD1: PCR (SB1640- SB1623), labeling: SB1623. SNR13: PCR(SB1309-hm427), labeling, hm427 or SB1310. SNR33: PCR(SB1319-1322), label with SB1322 . Sen1: BamH1 fragment out of pRS416 +-700 Sen1, label with hm331. sCR1: PCR with (sCR1 up and down), label with sCR1 down. Ssu72: PCR: hm550, 551, labeling hm551.

#### NET-seq

W303 bar1Δ Rpb3::Flag cells were grown in minimal complete medium and harvested at a density of 5x10^7^ cells/ml (asynchronous sample, 1320 ml) or arrested with 0.15 μg/ml αF (5x10^7^ cells/ml for 2.5 hr) or 15 μg/ml nocodazole (3x10^7^ cells/ml for 2 hr.). For Vector and Sen1 samples, W303 bar1Δ Rpb3::Flag cells were transformed with pGSen1Myc or pYMHHM, grown in selective minimal media in raffinose and induced for 3hrs with 2% galactose. Cells were harvested through filtration and frozen biomass disrupted in a mixer mill for 15 min at 15 Hz in 5 3-min intervals.

NET-seq conditions, immunoprecipitations, isolation of nascent RNA, and library construction were carried out as previously described (Churchman and Weissman, 2012), with the following modifications. Ligation of adapters was done directly to the 3′ end of isolated nascent RNA. A random hexamer sequence was added to the linker to improve ligation efficiency and allow for the removal of any library biases generated from the RT step as described in Mayer et al. (Mayer et al., 2015). After library construction the size distribution of the library was determined by using a 2100 Bioanalyzer (Agilent) and library concentrations were determined by Qubit 2.0 fluorometer (Invitrogen). 3′ end sequencing of all samples was carried out on an Illumina NextSeq 500 with a read length of 75.

#### Sequencing data alignment

NET-seq reads were aligned as follows. The adaptor sequence (ATCTCGTATGCCGTCTTCTGCTTG) was removed from all reads using cutadapt with the following parameters: -O 3 -m 1–length-tag ‘length = ‘. Raw fastq files were filtered using PrinSeq (http://prinseq.sourceforge.net/) with the following parameters: -no_qual_header -min_len 7 -min_qual_mean 20 -trim_right 1 -trim_ns_right 1 -trim_qual_right 20 -trim_qual_type mean -trim_qual_window 5 -trim_qual_step 1. Random hexamer linker sequences (the first 6 nucleotides at the 5′ end of the read) were removed using custom python scripts but remained associated with the read and reads were then aligned to the SacCer3 genome obtained from the *Saccharomyces* Genome Database using the TopHat2 aligner with the following parameters:–read-mismatches 3–read-gap-length 2–read-edit-dist 3–min-anchor-length 8–splice-mismatches 1–min-intron-length 50–max-intron-length 1200–max-insertion-length 3–max-deletion-length 3–num-threads 4–max-multihits 100–library-type fr-firststrand–segment-mismatches 3–no-coverage-search–segment-length 20–min-coverage-intron 50–max-coverage-intron 100000–min-segment-intron 50–max-segment-intron 500000–b2-sensitive. To avoid any bias toward favoring annotated regions the alignment was performed without providing a transcriptome. Reverse transcription mispriming events are identified and removed where molecular barcode sequences correspond exactly to the genomic sequence adjacent to the aligned read. For NET-seq only the position corresponding to the 5′ end of the sequencing read (after removal of the barcode), which corresponds to the 3′ end of the nascent RNA fragment, is recorded with a custom python script using HTSeq package (Anders et al., 2015).

#### Gene expression analysis

For gene expression analysis each dataset was first normalized by the number of 10^6^ uniquely mapped reads. The reads per gene per million mapped reads (RPM) were calculated for genes that were expressed in at least one of the samples being compared. To allow comparison of genes that were expressed in only one sample genes with 0 reads were given a pseudo-count of 0.1. Gene expression was then compared by plotting the log2 RPM for each sample. Annotations for coding genes were derived from Pelechano et al. (2013) by taking the major transcript isoform for each gene. CUT and SUT annotations obtained from Xu et al. (2009). Cumulative distribution functions for differences in gene expression were calculated by taking the log2 ratio of expression for each gene in one sample compared to another.

#### Average profile analysis around the TSS and polyadenylation site ‘aggregrate plots’

NET-seq reads around the TSS and polyadenylation sites are calculated for non-overlapping genes in 1bp bins using the deepTools program (Ramírez et al., 2014). Annotation for TSS and pA sites were derived from (Pelechano et al., 2013) by taking the major transcript isoform for each gene. The TSS and pA average profiles were calculated using non-overlapping protein coding genes with an RPKM greater than 10 in the empty vector NET-seq data and that are at least 500 bp long (N = 2792). TSS and 3′ end profiles for CUTs and SUTs were calculated using all annotated CUTs and SUTs from Xu et al. (Xu et al., 2009). Data for each plot are normalized as follows. First, each NET-seq library is normalized by the number of million uniquely mapped reads. NET-seq data for each gene used in the average profile is then normalized by summing the total number of reads for that gene and dividing by the length of the window analyzed. Each position is then normalized by average density value for that gene, thereby equalising the contribution from lowly and highly expressed genes. For TSS analysis this length is 1100 and for pA analysis 550. After each gene is normalized the average profile and 95% confidence interval are calculated, using a 25 base pair sliding window, which results in average Pol II density.

#### Pausing analysis

Pause detection in the NET-seq data was determined as described in Churchman and Weissman (2011). Briefly, the a site was considered a pause if the Pol II density at that nucleotide was at least three standard deviations above the mean of a sliding window of 200 bp around that position. To be considered for pause analysis a position must have at least four normalized reads when NET-seq data are normalized by10^6^ uniquely mapped reads. Pause sites were determined for the same subset of genes used to calculate the average Pol II profiles. To compare the pause density of regions near the promoter versus the gene body region the sum of the pause density in the first 300 bp downstream of the TSS (promoter region) was compared to the sum of the pause density from 300 bp downstream of the TSS to the pA site of that gene (body). The ratio of pause density in the gene body was then compared to the pause density near the promoter.

#### Termination efficiency

NET-seq reads for a region ± 100bp around pA sites were quantified for all genes ≥ 200bp long and no overlap with other transcripts at least 100 nt of TIF-seq end on same strand. Termination ratios (−100 to-50 upstream/50 to 100 downstream read count) were then plotted as boxplot using R.

### Quantification and Statistical Analysis

Data was quantified using ImageJ or AIDA image analysis software and normalized to an internal control. Details to statistical methods including number of replicates (n) are specified in the figure legends. Significance was calculated using Student’s t test, Fisher’s exact test and MEME (multiple expectation maximization for motif elicitation).

### Data and Software Availability

The accession number for the raw and processed NET-seq data reported in this paper is GEO: GSE86419. The raw data reported in this paper has been deposited to Mendeley: https://doi.org/10.17632/bsrvhwgs5j.1

## Supplemental Information

Document S1. Figures S1–S6 and Table S1

Document S2. Article plus Supplemental Information
