# Supplementary material for: Cell-Cycle Modulation of Transcription Termination Factor Sen1
Source: Mol Cell. 2018 Apr 19;70(2):312–326.e7. doi: 10.1016/j.molcel.2018.03.010 (PMC5919780; doi:10.1016/j.molcel.2018.03.010)
Supplement: Document S1. Figures S1–S6 and Table S1 [file mmc1.pdf]

Molecular Cell, Volume *70*

Supplemental Information

Cell-Cycle Modulation

of Transcription Termination Factor Sen1

Hannah E. Mischo, Yujin Chun, Kevin M. Harlen, Brendan M. Smalec, Somdutta Dhir, L. Stirling Churchman, and Stephen Buratowski

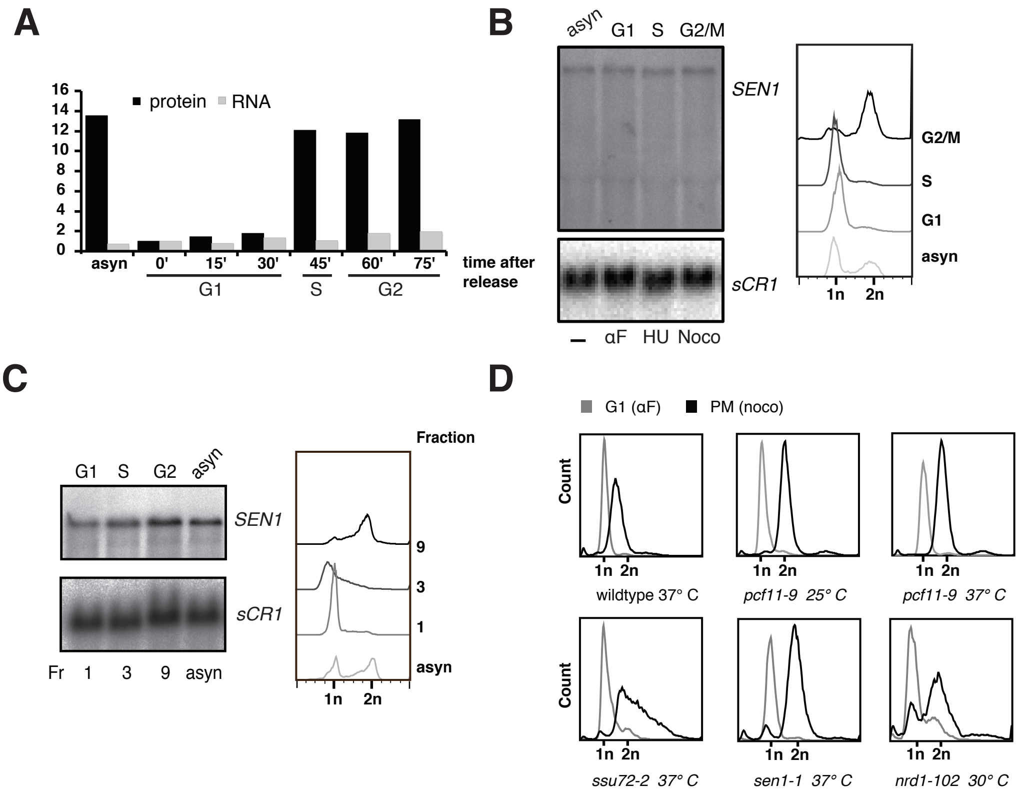

Figure S1, related to Figure 1:

A) Quantification of Figure 1A and B. Levels were normalised to levels at time of α-Factor release (0').

B) 10 µg RNA from arrested wild type cells (as in Figure 1C) was analysed by RNA blot for expression of *SEN1* and *sCR1*.

C) 11 µg RNA from elutriated cells (as in Figure 1D) was analysed by RNA blot for expression of *SEN1* and *sCR1*.

D) FACS profiles for cells analysed by RNA Blot in Figure 1E.

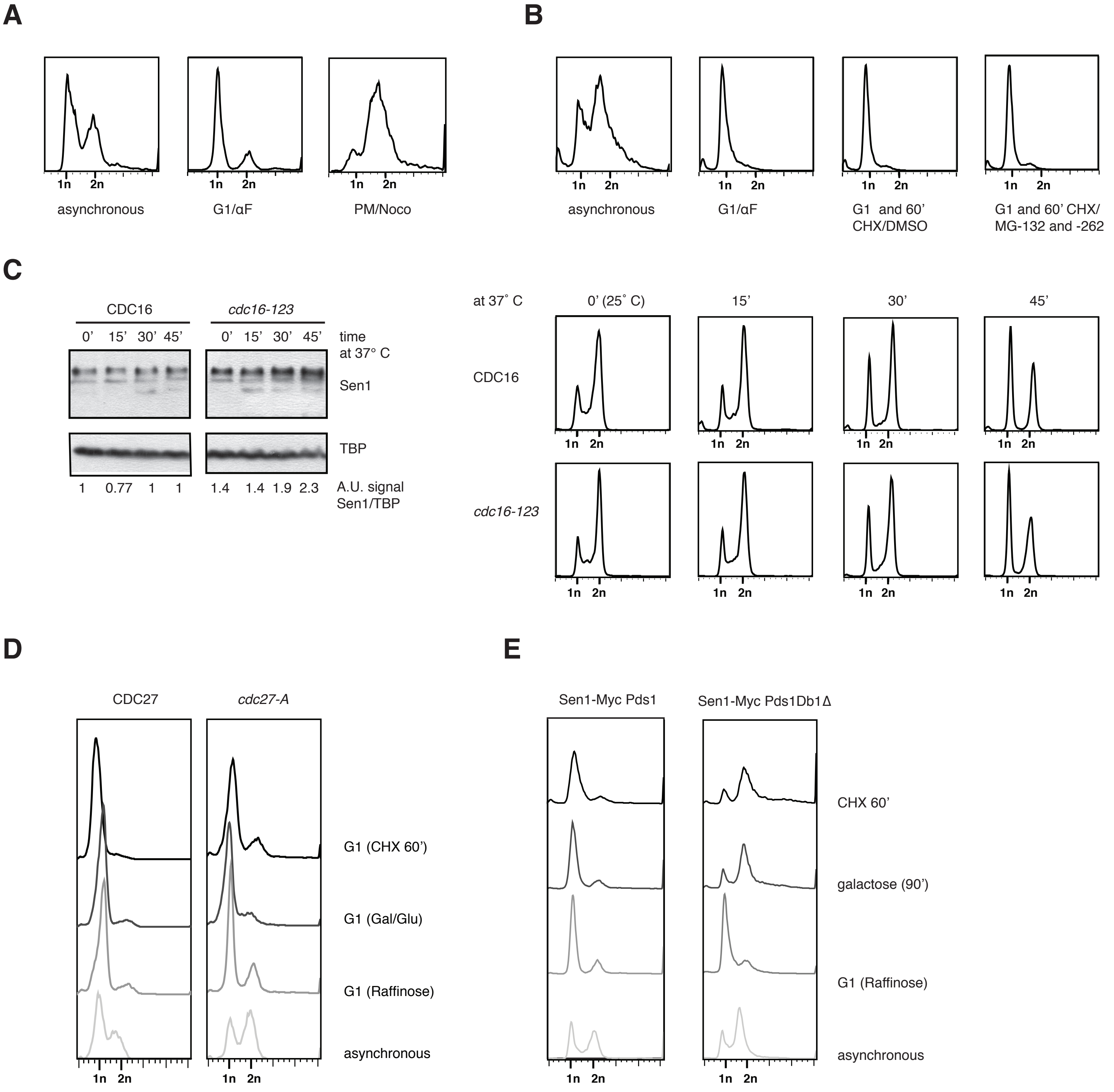

Figure S2, related to Figure 2:

A) FACS analysis of cells analysed in Figure 2A (asynchronous; cells before experiment, G1/PM; cells immediately before CHX addition).

B) FACS analysis of cells analysed in Figure 2B.

C) Sen1 accumulates in the temperature sensitive APC mutant *cdc16-123*. *cdc16-123* and wild type cells were grown in YPD at 25° C and shifted to 37° C for the indicated times. Untagged, endogenous Sen1 levels were analyzed from whole cell extracts and compared by immunoblotting to TBP (left panel), loading 2.1x107 cells/lane. Numbers under the lanes denote Sen1 signal intensity in A.U. normalized to TBP signal and to the 0 min time point of the wild type control.

D) FACS analysis of cells analysed in Figure 2C.

E) FACS analysis of cells analysed in Figure 2E.

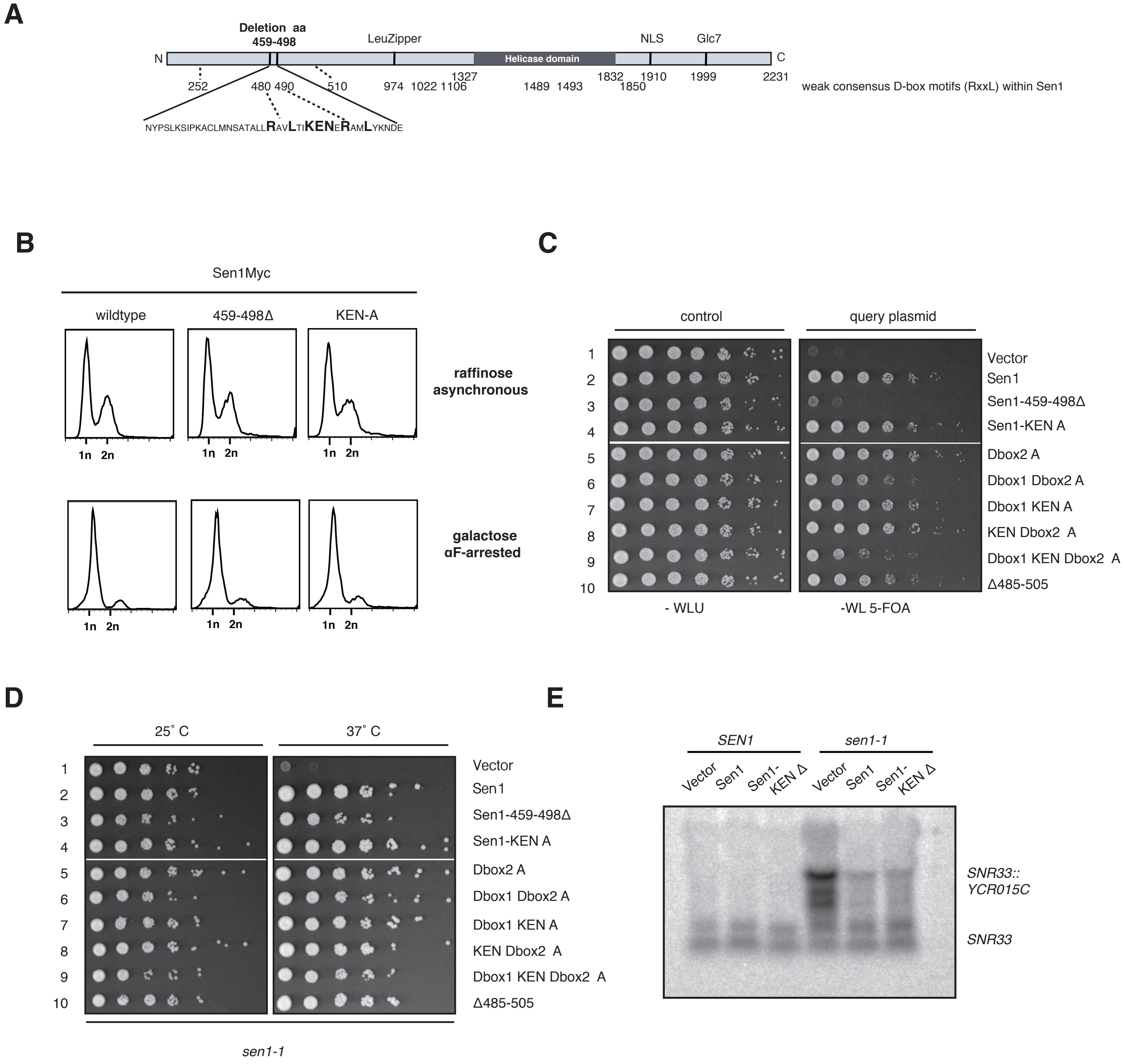

Figure S3, related to Figure 3:

A) Schematic model of Sen1 domain organization as in Figure 3A. Positions of the weak D-box consensus RxxL is indicated beneath the diagram.

B) FACS analysis of cells analysed in Figure 3B.

C) Five-fold serial dilutions (starting at 1.2x 105 cells/spot) of sen1Δ cells, carrying pRS416 +-700Sen1 and query plasmids 1-11 (left panel) or query plasmids alone (right panel). Query plasmids: 1, Vector; pRS414. 2, Sen1; pRS414+-700Sen1. 3-11 indicated mutant derivatives of pRS414+-Sen1.

D) Five-fold serial dilutions (as C) of SEN1 and sen1-1 cells transformed with centromeric plasmids pRS416 (Vector), and plasmids 1-11 all based on pRS416 +- 700 Sen1 and grown at 37° C.

E) RNA blot analysis (1% agarose) of 15 µg RNA isolated from SEN1 or sen1-1 cells transformed with Vector, pGSen1Myc (Sen1), or pGSen1Myc-459-498Δ (459-498Δ) and induced with galactose for 3 hrs at permissive temperature prior to a 30 min shift to non-permissive temperature. RNA blots were probed against SNR33.

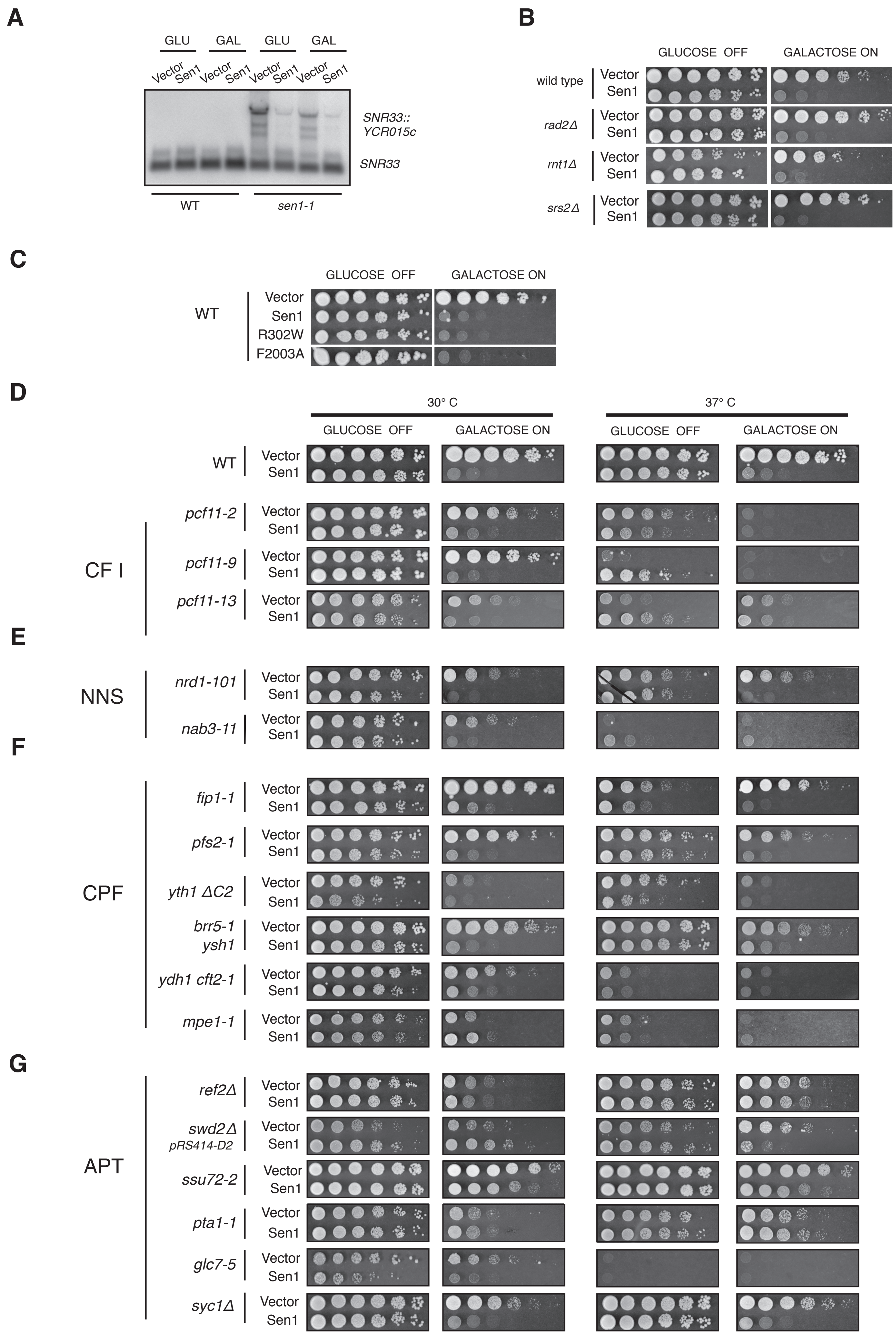
A
GLU
GAL
GLU
GAL
Vector
Sen1
Vector
Sen1
Vector
Sen1
Vector
Sen1
SNR33::
YCR015c
SNR33
WT
sen1-1
B
GLUCOSE OFF
GALACTOSE ON
wild type
rad2Δ
rnt1Δ
srs2Δ
C
WT
R302W
F2003A
D
30° C
37° C
pcf11-2
pcf11-9
pcf11-13
CF I
E
NNS
nrd1-101
nab3-11
F
CPF
fip1-1
pfs2-1
yth1 ΔC2
brr5-1
ysh1
ydh1 cft2-1
mpe1-1
G
APT
ref2Δ
swd2Δ
pRS414-D2
ssu72-2
pta1-1
glc7-5
syc1Δ

Figure S4, related to Figure 4:

A) RNA blot analysis of the SNR33 locus. See Figure 4A.

B) Serial five-fold dilutions of rnt1Δ, rad2Δ, srs2Δ and wild type cells transformed with Vector, pGSen1Myc, or the point mutations F2003A and R302W introduced into pGSen1Myc (pGSM-F2003A and pGSM-R302W). Growth was observed at 30° C.

C) Serial five-fold dilutions of mutants of CFI component Pcf11. pcf11-2 has defective cleavage activity and consequently defects in mRNA termination, pcf11-13 has a mutation in the CID and affects snoRNA termination, whereas pcf11-9 carries mutations that affect both processes (Grant et al., 2011; Sadowski et al., 2003).
Cell growth observed at 30° (semi-permissive) and 37° C (non-permissive temperature).

E) Serial five-fold dilutions of the NNS component mutants nrd1-101 and nab3-11 starting at a cell density of 12 104 cells/spot.

F) Serial five-fold dilutions of mutants of CPF components.

G) Serial five-fold dilutions of mutants of APT components. SWD2 deletion is only viable in presence of Sen1 aa 1890-2092 containing a Glc7 interaction site and is therefore maintained transformed with pRS414D2 (pRS414 +-700 Sen1 D2) (Nedea et al., 2008).

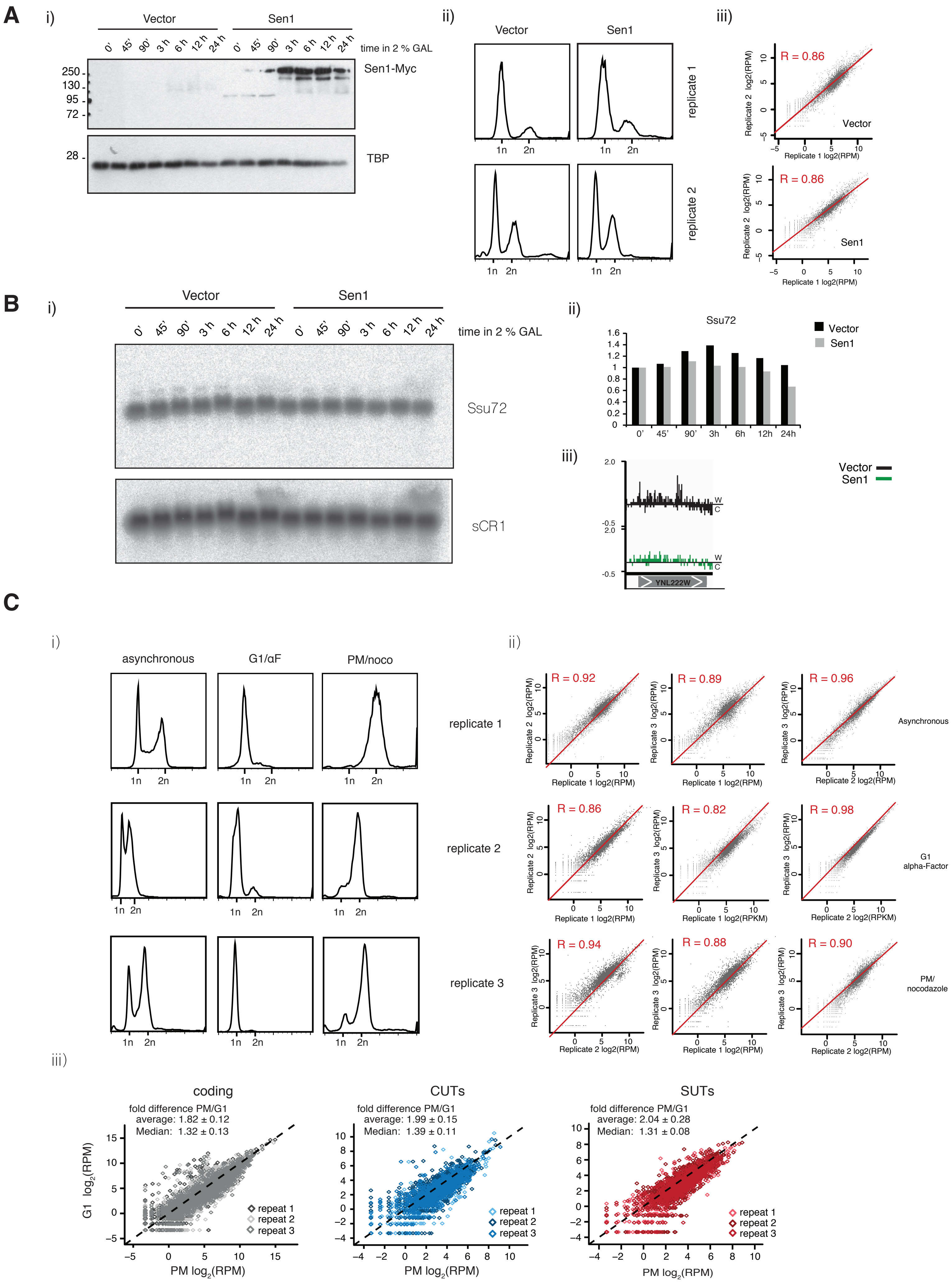

A
i)
Vector
Sen1
0' 45' 90' 3 h 6 h 12 h 24 h 0' 45' 90' 3 h 6 h 12 h 24 h
time in 2 % GAL
250
130
95
72
Sen1-Myc
28
TBP
ii)
Vector
Sen1
replicate 1
replicate 2
1n 2n
iii)
R = 0.86
Vector
Sen1
Replicate 2 log2(RPM)
Replicate 1 log2(RPM)
B
i)
Vector
Sen1
0' 45' 90' 3 h 6 h 12 h 24 h 0' 45' 90' 3 h 6 h 12 h 24 h
time in 2 % GAL
Ssu72
sCR1
ii)
Ssu72
Vector
Sen1
1.6
1.4
1.2
1
0.8
0.6
0.4
0.2
0
0' 45' 90' 3h 6h 12h 24h
iii)
2.0
-0.5
W
C
YNL222W
Vector
Sen1
C
i)
asynchronous
G1/αF
PM/noco
replicate 1
replicate 2
replicate 3
1n 2n
ii)
R = 0.92
R = 0.89
R = 0.96
R = 0.86
R = 0.82
R = 0.98
R = 0.94
R = 0.88
R = 0.90
Asynchronous
G1
alpha-Factor
PM/
nocodazole
Replicate 1 log2(RPM)
Replicate 2 log2(RPM)
Replicate 3 log2(RPM)
Replicate 1 log2(RPKM)
Replicate 2 log2(RPKM)
iii)
coding
fold difference PM/G1
average: 1.82 ± 0.12
Median: 1.32 ± 0.13
CUTs
fold difference PM/G1
average: 1.99 ± 0.15
Median: 1.39 ± 0.11
SUTs
fold difference PM/G1
average: 2.04 ± 0.28
Median: 1.31 ± 0.08
G1 log2(RPM)
PM log2(RPM)
repeat 1
repeat 2
repeat 3

Figure S5, related to Figure 6:

A.i) Immunoblot of Sen1 expression in cells transformed with Vector or pGSen1Myc and induced for indicated times. Whole cell TCA extracts from approximately $7*10^6$ cells per lane.

A.ii) FACS analysis of Vector or Sen1 expressing cells used in the NETseq analysis. Due to slow growth in minimal media supplemented with raffinose and galactose, cells are mostly found in G1.

A.iii) Scatterplots of Log_2(RPM) values of replicates 1 and 2 for Sen1 and Vector. R denotes the Pearson correlation coefficient of the respective RPM values.

B.i) 15µg RNA from cells isolated in parallel with protein extracts described in Figure S6A.i, was separated on a 1 % agarose gel, transferred onto nitrocellulose and probed against *SSU72*.

B.ii) Quantification of Northern Blot signals relative to *sCR1* and 0 minutes induction.

B.iii) NET-seq profiles for Ssu72 (*YNR222w*) for Vector (black) and Sen1 (green) samples. RPM values for Ssu72 are 65.9 (Vector) and 25.6 (Sen1).

C.i) FACS analysis of asynchronous, G1- (αF) or G2/M- (noco) arrested cells used in the NETseq analysis.

C.ii) Scatterplots of Log_2(RPM) values of replicates 1-3 for asynchronous, αF and nocodazole arrested cells. R denotes the Pearson correlation coefficient of the respective RPM values.

C.iii) RPM values for coding (grey, rep1, n=6490; rep2,3 n= 6594), CUTs (blue, n = 924) and SUTs (red, rep1, n=834; rep2,3, n=845) in G1- versus G2-arrested cells depicted as scatterplot.

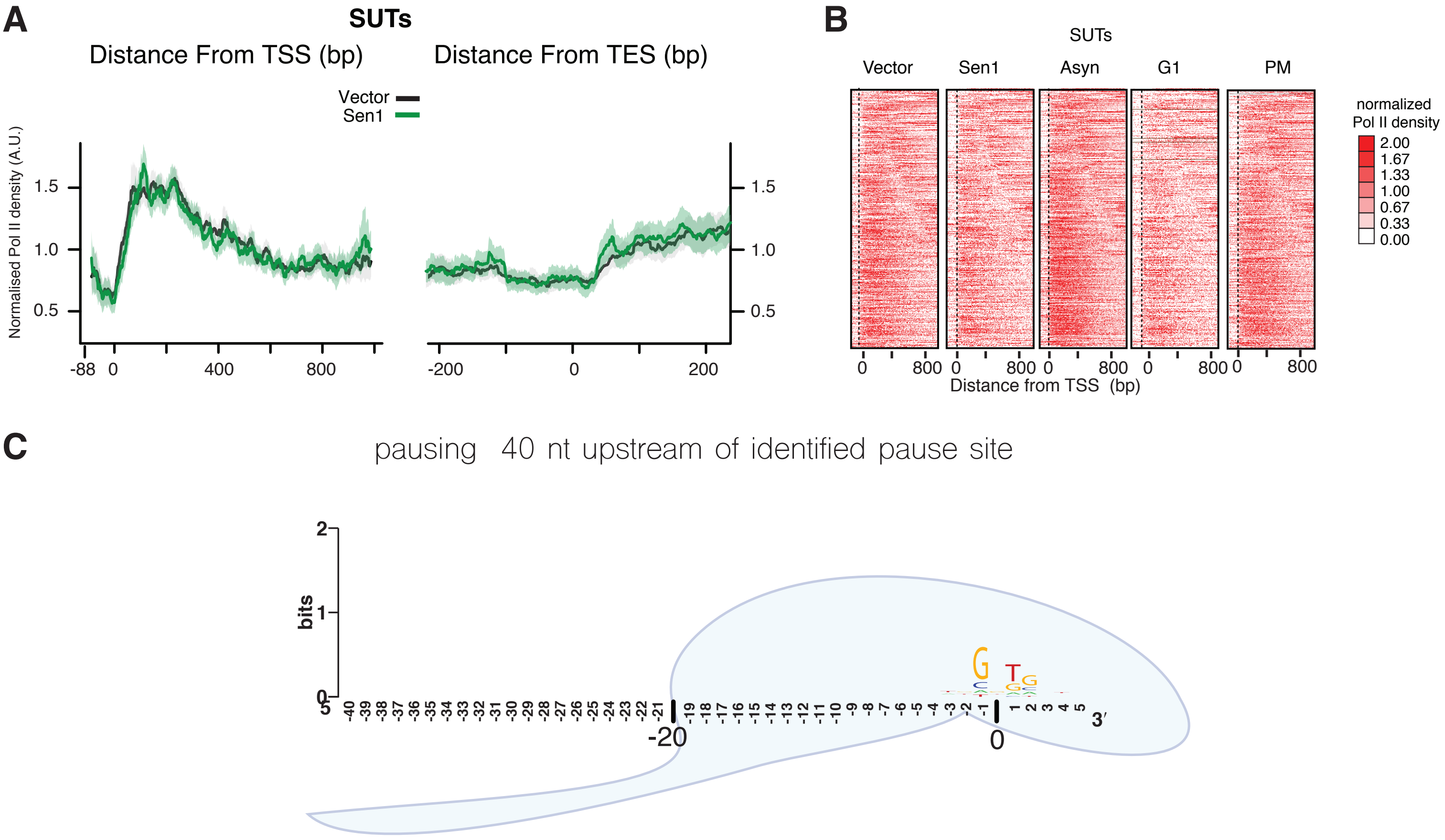

Figure S6, related to Figure 7:

A) Aggregate plot of all SUTs with an RPKM >10 (n= 847) anchored at the transcription start site (TSS) or the annotated transcription end site (TES).

B) Heatmap of all SUTs in Vector or Sen1-expressing cells, as well as asynchronous and G1 arrested cells.

C) MEME search of 40 nt upstream of the pause sites identifies no additional motif.

Table S1, related to Key Ressources Table.: Plasmids used in this study

Name	Backbone	Features	Construction	Reference
pYMHHM	pYES (AmpR, 2μ,URA3)	5xMyc-2xTEV-6xHis-MCS-6xHis-2xTEV-5xMyc		Genescript
pGSen1Myc	pYMHHM	BamH1-*SEN1*-Not1-6xHis-2xTEV-5xMyc	Codon optimized Sen1 sequence inserted into pYMHHM	Geneart
pGSM-F2003A	pGSen1Myc	Point mutation F2003A	SDM amplified with hm527	This study
pGSM-R302W	pGSen1Myc	Point mutation R302W	SDM amplified with Hm529	This study
pGSM-K1363A	pGSen1Myc	Point mutation K1363A	SDM amplified with hm510	This study
pGSM-D1590A	pGSen1Myc	Point mutation D1590A	SDM amplified with Hm514	This study
pGSen1Myc-459-498Δ	pGSen1Myc	Deletion of 30 aa around KEN-box	PPL: pGSen1Myc amplified with hm493 and hm492	This study
pRS416 +-700 Sen1	pRS416 (AmpR, CEN, URA3)	Sen1 with 700 nt endogenous promoter and terminator sequence	Sen1 +flanking region amplified with hm456 and hm457, subcloned into Topo and transferred into pRS416	This study
pRS414+-700 Sen1	pRS414 (AmpR, CEN, TRP1)	As pRS416 +-700 Sen1	As pRS416 +-700 Sen1	This study
pRS414 +-700 Sen1-459-498Δ	pRS414 +-700 Sen1	30aa deletion around KEN-box	PPL: pRS414+-700Sen1 amplified with hm480 hm477.	This study
pRS414 +-700 Sen1 D2	pRS414 (AmpR, CEN, TRP1)	aa 689-2231 of Sen1	PPL: pRS414+-700Sen1 amplified with hm472, hm473	This study
pRS414 +-700 Sen1-KEN A	pRS414 (AmpR, CEN, TRP1)	aa K486A, E487A,N488A	PPL: pRS414+-700Sen1 amplified with 3 and 6	This study
pRS414+-700 Sen1-Dbox2 A	pRS414 (AmpR, CEN, TRP1)	aa R490A, L493A	PPL: pRS414+-700Sen1 amplified with 3 and 5	This study
pRS414+-700 Sen1-Dbox1 Dbox2 A	pRS414 (AmpR, CEN, TRP1)	aa R480A, L483A, R490A, L493A	PPL: pRS414+-700Sen1 amplified with 1 and 5	This study
pRS414+-700 Sen1-Dbox1 KEN A	pRS414 (AmpR, CEN, TRP1)	aa R480A, L483A, K486A, E487A, N488A	PPL: pRS414+-700Sen1 amplified with 1 and 6	This study
pRS414+-700 Sen1-KEN Dbox 2 A	pRS414 (AmpR, CEN, TRP1)	aa K486A, E487A, N488A, R490A, L493A	PPL: pRS414+-700Sen1 amplified with 3 and 4	This study
pRS414+-700 Sen1-Dbox1 KEN Dbox 2 A	pRS414 (AmpR, CEN, TRP1)	aa R480A, L483A, K486A, E487A, N488A, R490A, L493A	PPL: pRS414+-700Sen1 amplified with 1 and 4	This study
pRS414+-700 Sen1-Δ485-505	pRS414 (AmpR, CEN, TRP1)	Deletion of aa 485-505 comprising KEN and Dbox2.		This study
P258	YIplac211/Gal1-10	PDS1Δ db, which deletes the destruction box of Pds1 under control of pGAL1-10		Frank Stegmeier/A Amon lab.

SDM: site directed mutagenesis
PPL: phosphorylated primer extension and ligation
